# Phase I study of CAR-T cells with PD-1 and TCR disruption in mesothelin-positive solid tumors

**DOI:** 10.1038/s41423-021-00749-x

**Published:** 2021-08-11

**Authors:** Zhenguang Wang, Na Li, Kaichao Feng, Meixia Chen, Yan Zhang, Yang Liu, Qingming Yang, Jing Nie, Na Tang, Xingying Zhang, Chen Cheng, Lianjun Shen, Jiaping He, Xun Ye, Wei Cao, Haoyi Wang, Weidong Han

**Affiliations:** 1 Medical School of Chinese PLA, Beijing, China; 2 Department of Biotherapeutic, the First Medical Center, Chinese PLA General Hospital, Beijing, China; 3 State Key Laboratory of Stem Cell and Reproductive Biology, Institute of Zoology, Chinese Academy of Sciences, Beijing, China; 4 School of Life Sciences, University of Science and Technology of China, Hefei, China; 5 Gracell Biotechnologies (Shanghai) Co., Ltd, Shanghai, China; 6 Institute for Stem Cell and Regeneration, Chinese Academy of Sciences, Beijing, China

**Keywords:** CAR-T, CRISPR-Cas9, PD-1, TCR, Mesothelin, Tumour immunology, Cancer therapy

## Abstract

Programmed cell death protein-1 (PD-1)-mediated immunosuppression has been proposed to contribute to the limited clinical efficacy of chimeric antigen receptor T (CAR-T) cells in solid tumors. We generated PD-1 and T cell receptor (TCR) deficient mesothelin-specific CAR-T (MPTK-CAR-T) cells using CRISPR-Cas9 technology and evaluated them in a dose-escalation study. A total of 15 patients received one or more infusions of MPTK-CAR-T cells without prior lymphodepletion. No dose-limiting toxicity or unexpected adverse events were observed in any of the 15 patients. The best overall response was stable disease (2/15 patients). Circulating MPTK-CAR-T cells peaked at days 7–14 and became undetectable beyond 1 month. TCR-positive CAR-T cells rather than TCR-negative CAR-T cells were predominantly detected in effusion or peripheral blood from three patients after infusion. We further confirmed the reduced persistence of TCR-deficient CAR-T cells in animal models. Our results establish the preliminary feasibility and safety of CRISPR-engineered CAR-T cells with PD-1 disruption and suggest that the natural TCR plays an important role in the persistence of CAR-T cells when treating solid tumors.

## Introduction

Mesothelin (MSLN) is an attractive target for cancer immunotherapy because it is highly expressed in a broad spectrum of solid tumors but present at low levels on normal tissues such as peritoneal, pleural, and pericardial mesothelial surfaces [1]. Clinical trials of MSLN-targeted chimeric antigen receptor T (CAR-T) cells for the treatment of solid tumors have shown that this treatment is safe but has very limited efficacy [2, 3].

Programmed cell death protein-1 (PD-1), a key immune checkpoint receptor, is upregulated in T cells following antigen encounter. It mediates immunosuppression when engaged by its ligand, programmed cell death ligand 1 (PD-L1), which is predominantly expressed in the tumor microenvironment [4]. Recently, PD-1 and PD-L1 blockade using monoclonal antibodies has resulted in unprecedented clinical efficacy in patients with a variety of cancers [5]. PD-1-mediated immunosuppression has been proposed to be involved in CAR-T cell hypofunction [6]. The combination of CAR-T cell therapy with anti-PD-1 antibody treatment has shown encouraging antitumor activity in patients [7, 8], suggesting that the PD-1 axis plays an important role in inhibiting CAR-T cell efficacy in the hostile solid tumor microenvironment. An alternative strategy to block the PD-1 axis in CAR-T cells is to disrupt cell-intrinsic PD-1 expression via clustered regularly interspaced short palindromic repeats (CRISPR) and the CRISPR-associated protein 9 (Cas9) system (CRISPR-Cas9), which has resulted in enhanced antitumor activity in preclinical studies [9, 10].

Notably, the PD-1 axis blockade is a double-edged sword. The PD-1/PD-L1 pathway has been found to be involved in the modulation of both central and peripheral tolerance [11] and elimination of either pathway can result in the breakdown of tolerance and the development of autoimmunity, as reported in the anti-PD-1 antibody experience [12]. In terms of CAR-T cells with genetic disruption of PD-1, there could be an increased risk of autoimmunity due to breakdown of peripheral immune tolerance for the following reasons: (1) the threshold for T cell receptor (TCR)-mediated self-reactive T cell activation might be lower due to stimulation received by engagement of the specific CAR antigen and (2) self-reactive T cells could be expanded to a greater level as a consequence of CAR-mediated expansion, worsening autoimmune activity.

To enhance the antitumor activity and reduce the risk of potential autoimmune response, we used CRISPR-Cas9 technology in combination with lentiviral transduction to produce MSLN-directed 28ζ CAR-T cells with PD-1 and TCR disruption (MPTK-CAR-T cells). A single-chain variable fragment (P4 scFv) of a fully human antibody [13] was selected to construct the anti-MSLN CAR, termed the P4 CAR, in an effort to address potential CAR transgene immunogenicity. Considering the lack of clinical experience, particularly the safety data of CAR-T cells with PD-1 deletion, we initiated a dose-escalation study with a low initial dose (1–2 × 10^5^/kg) to assess MPTK-CAR-T cells in patients with MSLN+ advanced solid tumors. Here, we report on the safety, cellular kinetics, and antitumor activity of the infused MPTK-CAR-T cells.

## Materials and methods

### Preclinical section

#### Preclinical MPTK-CAR-T cell manufacturing

Peripheral blood mononuclear cells (PBMCs) were separated from healthy donor leukapheresis products using density gradient centrifugation at day 0. T cells were sorted from PBMCs with anti-CD4/CD8 magnetic beads (Miltenyi). Purified T cells were activated with anti-CD3/anti-CD28 Dynabeads for 48 h and then electroporated with Cas9 protein and the intended sgRNAs targeting *TCRα subunit constant* (*TRAC)* and *PDCD1*, as shown in Supplementary Fig. 1A, followed by transduction with a lentiviral vector that encoded the P4 CAR (which was constructed using the P4 scFv derived from a fully human anti-MSLN antibody [13], the hinge region from CD8α, the transmembrane and costimulatory regions from CD28, and the CD3ζ signaling region [14]). Transduced T cells were cultured and expanded in T cell culture medium mainly comprising X-VIVO medium (Lonza) plus 5% fetal bovine serum (FBS) (Gibco) in the presence of 300 IU/mL interleukin-2 (IL-2) for 6–8 days and then harvested for preclinical assays.

#### Assessment of frequency of gene editing

DNA was extracted from the control samples and gene-edited CAR-T cell products. Sequencing data derived from the areas around the gene-edited region were analyzed with the inference of the CRISPR edits (ICE) method, an improved algorithm of the tracking indels by decomposition (TIDE) method (https://ice.synthego.com) [15, 16].

#### Evaluation of the off-target sites of *TRAC and PDCD1* sgRNAs

The off-target sites were detected using Digenome-seq analysis [17]. The top 10 targets and top 3 targets with exons for each sgRNA were amplified by PCR and subjected to Sanger sequencing. The sequencing results were analyzed using the ICE method.

#### Flow cytometry

Harvested cells were stained for 20 min in the dark, washed twice in PBS, and analyzed using CytoFLEX (Beckman Coulter Inc.). The antibodies used are as follows: CD3-Pacific blue (300329), TCR-APC (306718), CD4-APC (317416), CD8-APC (301014), CD8-Brilliant Violet (301035), CD45RO-PE (304206), CCR7-Percp (353242) (all from Biolegend) and goat anti-human IgG (H+L)-AlexaFluor 647 (109-606-003) (Jackson ImmunoResearch).

#### Luciferase-based cytolysis assay and cytokine enzyme-linked immunosorbent assay (ELISA)

The CRL5826 cell line was obtained from ATCC, and the CRL5826-PD-L1-luci cell line was generated by transducing CRL5826 cells with lentivirus encoding the PD-L1 and luciferase genes. Cancer cells were suspended at 1 × 10^5^ cells/mL in RPMI 1640 medium and cocultured with CAR-T cells at the indicated E:T ratios at 37 °C with 5% CO_2_. At the indicated incubation time, substrate (Promega) was added, and the luminescence was determined by PerkinElmer VICTOR X3. The results are reported as the percentage of killing based on the luciferase activity of the remaining tumor cells (% killing = 100−((RLU from well with effector and target cell coculture)/(RLU from well with target cells)×100)). Supernatants were harvested, and cytokines (interferon-γ (IFN-γ) and IL-2) produced by CAR-T cells were measured by ELISA kits (Biolegend).

#### Repetitive tumor challenge assay

CAR-T cells were cocultured with CRL5826-PD-L1 cells at a 2:1 E:T ratio. Every 2 days, CAR-T cells were counted, and new tumor cells were added at a constant E:T ratio of 2:1. In each round, the number of CAR-T cells was counted, and the proportion of CD3-positive cells and TCR-positive cells was detected by flow cytometry.

#### Animal model and in vivo CAR-T cell function detection

Six-week-old female NOD-Prkdc scid Il2rgnull (NPG) mice (Vitalstar) inoculated subcutaneously with 2 × 10^6^ CRL5826-PD-L1-luci tumor cells as a CRL5826-PD-L1 cell-line-derived xenograft (CDX) model or engrafted with pancreatic carcinoma patient-derived xenografts (PDXs, Vitalstar) were randomly divided into different groups when the tumors were between 200 and 300 mm^3^ in volume. CAR-T cells were administered intratumorally or intravenously twice with a one-week interval at a dose of 5 × 10^6^ cells/mouse (the CAR+ cell percentage was 50%). Bioluminescence images, body weight, and tumor size were monitored weekly.

To detect the persistence of MPTK-CAR-T cells in the CRL5826-PD-L1 CDX model, 2 × 10^7^ CAR-T cells were injected intravenously. Bodyweight and tumor size were monitored weekly. Mouse peripheral blood (PB) was collected, and the proportions of human CD45+, CD3+, and TCR+ cells were analyzed.

### Clinical section

#### Study design and participants

This clinical study was a proof of concept, single-arm, open-label, dose-escalation study in which patients received an escalating dose of MPTK-CAR-T cells (0.1–9 × 10^6^ CAR-T cells/kg) with the following four dose levels: 0.1–0.2 × 10^6^ CAR-T cells/kg, 0.5–1.0 × 10^6^ CAR-T cells/kg, 1.5–3.0 × 10^6^ CAR-T cells/kg, and 4.5–9.0×10^6^ CAR-T cells/kg. A deviation of within ±20% was acceptable between the actual dose infused and the predefined dose level (DL). Lymphodepleting chemotherapy was not included in this protocol mainly for the following reasons: (1) concern that lymphodepleting chemotherapy would increase the risk of uncontrolled in vivo expansion and proliferation of CAR-T cells with PD-1 disruption, which could affect safety; and (2) lymphodepleting chemotherapy would put patients at risk for infection, it has shown limited antitumor activity in solid tumors, and the efficacy of MPTK-CAR-T cells in humans is unknown, suggesting that the lymphodepleting chemotherapy-related risks outweigh the benefits.

Patients were allowed to receive a second or third infusion of MPTK-CAR-T cells at the same or higher dose in the case of undetectable circulating MPTK-CAR-T cells and manageable toxicity following prior infusion. PB samples were obtained before MPTK-CAR-T cell infusion and at multiple predetermined time points after infusion to monitor the cellular kinetics of MPTK-CAR-T cells and cytokine production. Optional tumor lesion biopsies were collected at baseline and 2–4 weeks post-infusion. The study has been registered at ClinicalTrials.gov (NCT03545815).

We enrolled adult patients with measurable MSLN+ (≥10% of tumor cells expressing MSLN) locally advanced or metastatic solid tumors who failed at least 1 standard therapy or were unable to tolerate chemotherapy. The PD-L1 expression level in tumor tissue was not an inclusion criterion but was evaluated by immunohistochemistry in preinfusion biopsy specimens. An Eastern Cooperative Oncology Group (ECOG) performance status of 0–3 and essentially normal major organ function were required. Other therapies, including chemotherapy, targeted therapy, and radiotherapy or checkpoint blockade, must have been completed at least more than 1 month before MPTK-CAR-T cell infusion. Restaging was always carried out within one week before MPTK-CAR-T cell infusion. Patients with known infection with HIV, HBV, HCV, CMV, EBV, or HTLV were excluded. Patients with the active immune disease were excluded.

#### Study objectives and endpoints

The primary objectives were to characterize the safety and feasibility as well as in vivo expansion and persistence of MPTK-CAR-T cells in MSLN-expressing solid tumors. The secondary objective was to assess the disease response to MPTK-CAR-T cell infusion. Primary endpoints included dose-limiting toxicity (DLT), incidence and severity of MPTK-CAR-T cell-related adverse events (AEs), and CAR transgene copy numbers in PB within 4 weeks after the first infusion. Secondary endpoints included best overall response (BOR), disease control rate (DCR), and progression-free survival (PFS). BOR was defined as the best response recorded over the study for the individual subject in the study. When stable disease (SD) was believed to be the best response, a minimum duration of 4 weeks of SD must have been met. DCR was defined as the proportion of subjects with a BOR of complete response (CR), partial response (PR) or SD. PFS was defined as the time from the first MPTK-CAR-T cell infusion to the first objective documentation of tumor progression or death due to any cause. Subjects who did not progress or die or who started new anticancer treatment were censored on the date of last tumor assessment.

#### Safety assessments

All patients who received MPTK-CAR-T cells were evaluated for safety, which included the incidence and severity of MPTK-CAR-T cell infusion-related AEs. In addition to cytokine release syndrome (CRS), which was graded by the Upenn grading scale [18], other AEs were categorized according to the National Cancer Institute Common Terminology Criteria for AEs, version 5.0. A DLT was defined as any of the following MPTK-CAR-T cell-related (probable or definite) events occurring within the first 28 days after the first MPTK-CAR-T cell infusion: (1) any grade 3 or higher nonhematologic toxicity that was unresponsive (does not improve to <grade 3 toxicity) to appropriate treatment or had a >28-day duration; (2) grade 3–5 allergic reactions, or grade 2 allergic reactions in which symptoms reappeared after repeat infusion; and (3) grade 2–5 autoimmune reactions, including pericarditis, peritonitis, and pleuritis.

#### Evaluation of MSLN and PD-L1 expression by immunohistochemistry

Tissues from tumor biopsies were fixed in 10% formalin and paraffin-embedded. Sections were stained with hematoxylin and eosin or with anti-MSLN primary antibody (clone SP74, Abcam) and detected using goat anti-rabbit secondary antibody. Staining was detected using DAB, and slides were counterstained with hematoxylin. Brightfield images were acquired on a BX53 upright (Olympus) microscope. The process of assessing PD-L1 expression by immunohistochemistry was similar to that used to assess MSLN expression. An anti-PD-L1 primary antibody (clone ZR3, Gene Tech) was used for PD-L1 immunohistochemistry.

#### Clinical-grade MPTK-CAR-T cell manufacturing and infusion

PBMCs were separated from each patient’s leukapheresis product to prepare clinical-grade MPTK-CAR-T cells using density gradient centrifugation. Other manufacturing procedures were consistent with those of preclinical MPTK-CAR-T cell manufacturing, with the exception that no animal-derived serum was added to the culture medium, and clinical MPTK-CAR-T cells were cryopreserved after harvesting. MPTK-CAR-T cell infusion products were quality tested for number, purity, viability, sterility, transduction efficiency, and cytolytic activity prior to cryopreservation. Frozen clinical-grade MPTK-CAR-T cells were transported in vapor-phase liquid nitrogen to the infusion site, thawed at the bedside in a 37 °C water bath and intravenously infused immediately within 2 h after cell thawing.

#### Flow cytometric analysis of PD-1 and TCR expression in MPTK-CAR-T cell products

The anti-PD-1 antibody PD-1-BV421 (Biolegend, 329920) and anti-CD3 antibody CD3-FITC (BD Bioscience, 340542) were used for flow cytometric analysis of PD-1 and TCR expression in control cells and MPTK-CAR-T cell products. Control cells were derived from the same donor and had the same culture conditions as the MPTK-CAR-T cell products.

#### Assessment of the frequency of gene editing in MPTK-CAR-T cell products

The method used was the same as that described in the preclinical section.

#### Translocation detection

Translocation events between the *PDCD1* and *TRAC* loci were detected by quantitative PCR (qPCR) using a MightyAmp™ for Real-Time kit (Takara) and a LightCycler 480 (Roche). Four plasmids containing the possible translocation events were synthetized. The copy number standard curves were determined using the plasmids. Genomic DNA extracted from cell products from enrolled patients or healthy donors was prepared, and the copy number of each sample was detected by qPCR. The primers were as follows: forward primer for T1 GGGCACCCTCCCTTCAACCTGAC, reverse CCCATCCCCAGAAGGGCTCAGAAAT; forward primer for T2 CTGGGACAGTTTCCCTTCCG, reverse CTGCAGGGAGGTTTGCTCTC; forward primer for T3 CACAATACTGTTGGCCCTGGAAGAA, reverse CAAAGCCACACAGCTCAGGGTAA; forward primer for T4 AAGTAGGAGAGTTTGGTGGGCTCAG, reverse AAAGCCACACAGCTCAGGGTAAGG.

#### Detection of MPTK-CAR-T cells in patient samples

##### qPCR

Blood genomic DNA was extracted from 1 mL whole blood using the QIAamp DNA Blood Midi kit (Qiagen, 51185). Tissue genomic DNA was extracted from biopsy tissue using a DNeasy Blood & Tissue Kit (Qiagen, 69504). Genomic DNA was amplified using the TB Green Premix ExTaq (Tli RnaseH Plus) kit (Takara Biotechnology, RR420A). Amplification was detected in real-time using the Applied Biosystems 7500 Real-Time PCR System (Life Technologies). A primer pair targeting the WPRE region was used to characterize CAR copy number, while a primer pair targeting the housekeeping gene RPP30 was used as a control for cell number normalization. The primer pairs were experimentally validated with the following criteria: (i) a single gene-specific product was produced; (ii) the amplification efficiency ranged between 90% and 110%; and (iii) the cycle threshold (Ct) value of the no-template control was more than 40.

##### Flow cytometry

PB samples were collected in EDTA tubes after MPTK-CAR-T cell infusion for CAR-T cell analysis by flow cytometry on a FACSCanto II Plus platform (BD Biosciences). One hundred microliters of blood were stained with biotinylated human MSLN protein (ACROBiosystems, MSN-H826X) on ice for 30 min followed by streptavidin-APC (Invitrogen, SA1005), CD2-PE (BD Bioscience, 555635), CD45-V500 (BD Bioscience, 560777), CD3-FITC (BD Bioscience, 340542), CD4-PE/Cy7 (BD Bioscience, 557852), and CD8-APC/H7 (BD Bioscience, 560179) staining. PD-1 expression was checked with PD-1-BV421 (Biolegend, 329920) when necessary. Erythrocytes were lysed with red blood cell lysis solution (BD, 349202), and white blood cells were centrifuged and washed with 2 mL PBS before data acquisition.

##### RNAscope ISH

Tissues from tumor biopsies were fixed in 10% formalin and paraffin-embedded and cut into 5 μm sections. Then, RNAscope ISH was conducted according to the manufacturer’s protocol. Briefly, the sections were rehydrated in xylene and alcohol after baking at 60 °C for 1 h. Then, the sections were incubated with probes at 40 °C for 2 h after pretreatment with hydrogen peroxide, target retrieval reagent, and protease plus. The corresponding preamplifier, amplifier, and dyes were combined in turn with the specific probe. Then, images were taken after mounting the sections. To detect the infiltration of CAR-T cells into tumors, the RNAscope 2.5 HD Assay-RED kit, positive control probe PPIB, the DapB negative control probe, and the target scFv probe were used. The reagents used in these assays were all from Advanced Cell Diagnosis (ACD).

### Analysis of serum cytokines

After centrifugation, serum samples were aliquoted and frozen at −70 °C immediately. Cytokines were analyzed using a Luminex 200 apparatus (Luminex Corporation). The following cytokines, chemokines, and growth factors were measured in the cohort samples: IL-2, IL-6, IL-7, IL-8, IL-10, IL-12p70, IL-15, tumor necrosis factor-α (TNF-α), granulocyte-macrophage colony-stimulating factor (GM-CSF), IFN-γ, granzyme B, chemokine (C–C-motif) ligand 19 (CCL19), monocyte chemotactic protein-1 (MCP-1), von Willebrand factor A2 (vWF-A2), angiopoietin-1, and angiopoietin-2.

### Flow cytometric analysis of the TCR Vβ repertoire

TCR β-chain region variability was analyzed by flow cytometry by using a TCR Vβ Receptor kit (Beckman-Coulter, IM3497) as described [19, 20]. This kit is composed of 8 vials containing mixtures of conjugated TCR Vβ antibodies corresponding to 24 different specificities, which cover 70% of the normal human TCR Vβ repertoire.

### Statistics

Statistics were assessed and graphed with GraphPad Prism 7 software. Unpaired Student’s *t* test was applied for 2-group comparisons, and ANOVA with Tukey’s multiple-comparisons test was applied for comparing more than 2 groups. Data are represented as the mean ± standard deviation. *P* values of <0.05 were considered to be significant. The Mann–Whitney test was used to compare the mean peak expansion levels of circulating MPTK-CAR-T cells identified by qPCR within each dose group, and a *P* value <0.0083 (false-positive rate < 0.05) was considered statistically significant.

### Study approval

Animal experiments were approved by the Animal Ethics Committee of the Institute of Zoology, Chinese Academy of Sciences, and performed in accordance with institutional animal care and use guidelines. This clinical study was approved by the Institutional Review Board at Chinese PLA General Hospital. All research participants provided written informed consent prior to study participation at Chinese PLA General Hospital.

## Results

### Preclinical evaluation of MPTK-CAR-T cells

**Fig. 1:**
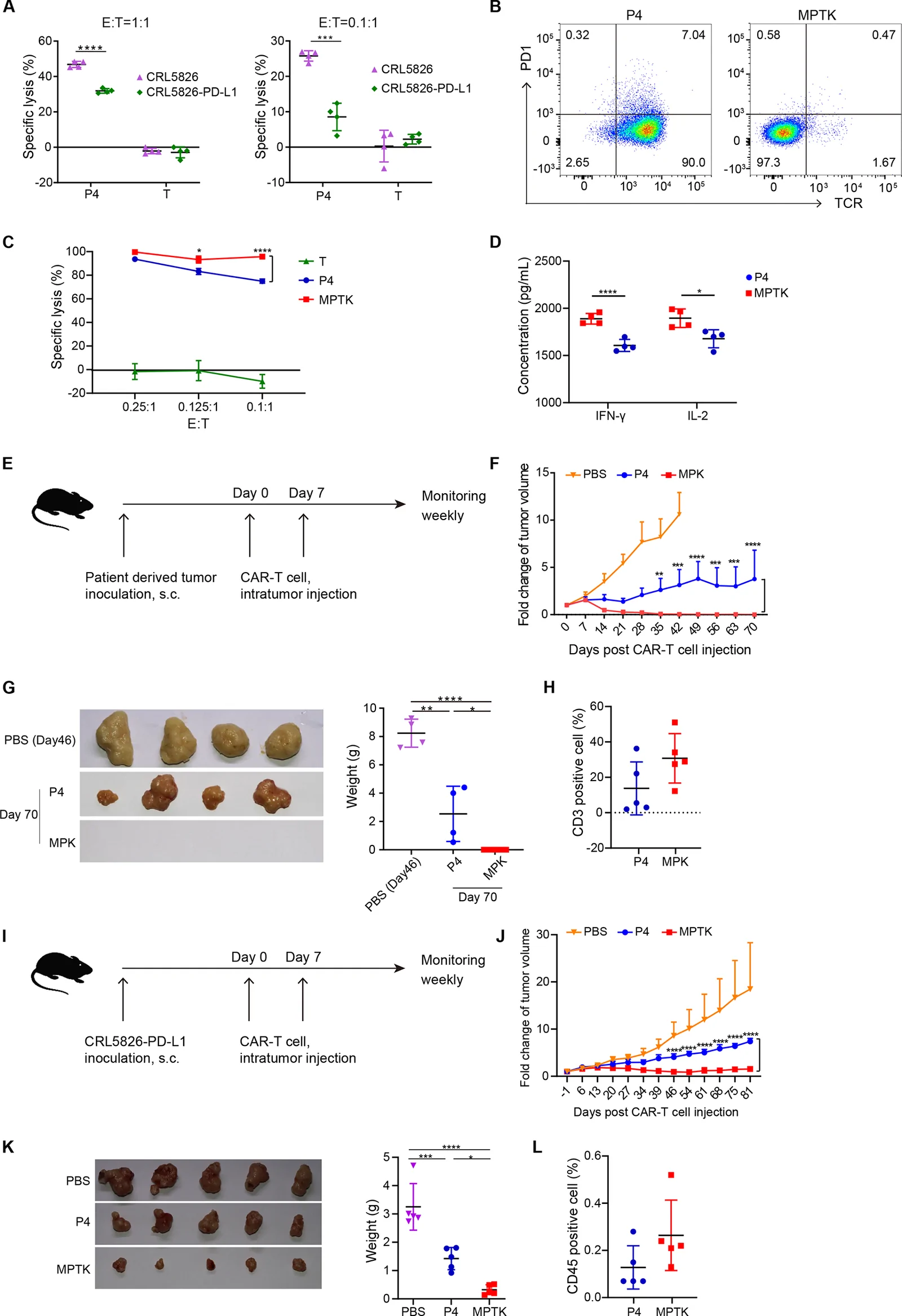
*PDCD1*/*TRAC* double knockout P4 CAR-T cells exhibited superior antitumor efficiency. **A** Bioluminescence-based cytotoxicity assay. The specific lysis of CRL5826 or CRL5826-PD-L1 tumor cells after being cocultured with P4 CAR-T cells at a 1:1 effector:target (E:T) ratio for 2 days or a 0.1:1 E:T ratio for 4 days is shown. T cells without lentivirus transduction are also shown. **B** Expression of PD-1 and TCR in P4 and MPTK-CAR-T cells by flow cytometry. **C** Cytotoxicity of P4 and MPTK-CAR-T cells cocultured with CRL5826-PD-L1 at a low E:T ratio for 3 days. **D** Production of the cytokines IFN-γ and IL-2 by MPTK and P4 CAR-T cells cocultured with CRL5826-PD-L1 cells at a 0.1:1 E/T ratio for 3 days. The essays in **A** and **B** were repeated independently using cells from three donors, and those in **C** and **D** were repeated using two donors. **E–H** Scheme of the in vivo assay to test the antitumor function of CAR-T cells. NPG mice were subcutaneously transplanted with patient-derived pancreatic tumor cells (PDX model). When tumors grew to ~200–300 mm^3^, MPK- or P4 CAR-T cells or PBS was injected intratumorally twice with 7 days between injections. The weights and tumor volumes of the mice were measured weekly (**E**). Tumor growth (**F**) and the sizes and weights of resected tumors (**G**) derived from PDX mice sacrificed at 46 days after CAR-T cell injection. **H** Peripheral blood analysis of the proportion of human CD3-positive cells at 42 days after CAR-T cell injection (*n* ≥ 4 animals per group). **I–L** Scheme of the in vivo assay to test the antitumor function of CAR-T cells. NPG mice were subcutaneously injected with CRL5826-PD-L1 cells (CDX model). When tumors grew to ~200–300 mm^3^, MPTK- or P4 CAR-T cells or PBS was injected intravenously twice with 7 days between injections. The weights and tumor volumes of the mice were measured weekly. **I** Tumor growth (**J**) and the sizes and weights of resected tumors (**K**) derived from CRL5826-PD-L1 tumor-bearing NPG mice sacrificed 81 days after CAR-T cell intravenous injection, and peripheral blood analysis of the proportion of human CD3-positive cells (**L**) at 69 days after CAR-T cell injection (*n* = 5 animals per group). Statistics were analyzed with unpaired Student’s *t* test (**A**, **D**, **H**, and **L**) and ANOVA with Tukey’s multiple-comparisons test (**C**, **F**, **G**, **J**, and **K**). Data are represented as the mean ± standard deviation (SD). *P < 0.05; **P < 0.01; ***P < 0.001; ****P < 0.0001

First, we assessed the MSLN-specific tumor lysis capability of P4 CAR-T cells (Supplementary Fig. 1B) using the CRL5826 cell line, which is both MSLN-positive and PD-L1-positive (Supplementary Fig. 1C). We also generated the CRL5826-PD-L1 cell line by inducing overexpression of PD-L1 to further augment PD-1/PD-L1 signaling (Supplementary Fig. 1C). At a 1:1 effector to target (E:T) ratio, P4 CAR-T cells lysed ~50% of CRL5826 cells after 2 days of incubation, while CRL5826-PD-L1 cells showed notable resistance to the cytolytic activity of CAR-T cells (Fig. 1A). This effect was also observed at a lower E:T ratio (0.1:1) with a longer incubation time (Fig. 1A). These results confirmed that the PD-1/PD-L1 pathway inhibits CAR-T cell function in vitro.

We next tested whether knocking out the *PDCD1* gene would enhance the antitumor effect of CAR-T cells. *PDCD1* knockout P4 (MPK-CAR-T) cells were generated via electroporation of Cas9-sgRNA ribonucleoprotein (RNP), and the knockout efficiency was ~80%, as quantified by ICE analysis [15, 16] (Supplementary Fig. 1A and D). MPK-CAR-T cells showed consistently higher cytotoxic efficacy at E:T ratios ranging from 0.2:1 to 0.00625:1 than P4 CAR-T cells (Supplementary Fig. 1E).

Considering that eliminating PD-1 might induce autoimmune responses, we knocked out the *TRAC* gene to reduce the potential risk. The knockout efficiencies of *PDCD1* and *TRAC* in MPTK-CAR-T cells were both ~90%, as quantified by ICE analysis and flow cytometry analysis (Supplementary Figs. 1A and 2A, and Fig. 1B). Knocking out *TRAC* via CRISPR-Cas9 led to the loss of both TCR and CD3 on the T cell surface, the flow cytometry signals of which were well correlated (Supplementary Fig. 2B). To evaluate the potential off-target effects of genome editing, we performed Digenome-seq [17] to predict the top off-target sites of each sgRNA and chose 10 genomic loci with the highest scores and the top three hits within exons for genotyping. Using ICE analysis, we genotyped all 26 sites in the MPTK-CAR-T cells (Supplementary Table 1) and did not detect any off-target mutations. No significant difference in CAR transduction efficiency (Supplementary Fig. 2C), proliferation (Supplementary Fig. 2D), CD4/CD8 ratio (Supplementary Fig. 2E), or T cell subsets (Supplementary Fig. 2F) was observed between P4 and MPTK-CAR-T cells. Consistent with the results for MPK-CAR-T cells, the MPTK-CAR-T cells showed higher cytotoxic efficacy at E:T ratios ranging from 0.25:1 to 0.1:1, and the MPTK-CAR-T cells had more cytokine secretion (Fig. 1C and D).

To evaluate the antitumor efficacy of gene-edited CAR-T cells in vivo, we used NPG mice (Vital Star) bearing CRL5826-PD-L1 tumors (CDX) and two patient-derived tumor xenograft (PDX) models derived from pancreatic cancer samples that were both MSLN positive (Supplementary Fig. 3A). In these models, MPK-CAR-T cells eradicated tumors rapidly and efficiently, while P4 CAR-T cells had lower efficacy in controlling tumor growth (Fig. 1E–G, and Supplementary Fig. 3B–G). The mouse PB samples indicated that MPK-CAR-T cells had slightly higher persistence than P4 CAR-T control cells (Fig. 1H and Supplementary Fig. 3H). Similarly, MPTK-CAR-T cells eradicated tumors faster and more efficiently than did P4 CAR-T cells (Fig. 1I, J and K) in the CRL5826-PD-L1 CDX model upon intravenous injection. When the tumor was nearly cleared by MPTK-CAR-T cells, at day 69 after CAR-T cell injection, we analyzed the PB samples from each group and observed a similarly low proportion of CAR-T cells, suggesting that no abnormal cell proliferation was induced by gene editing (Fig. 1L). These preclinical data demonstrate that eliminating *PDCD1* and *TRAC* by CRISPR/Cas9 technology improves the antitumor efficacy of CAR-T cells.

### Patients

Between March 2018 and January 2019, a total of 17 patients were enrolled, and 15 were treated with a total of 25 infusions of MPTK-CAR-T cells at an escalating dose (0.18–11 × 10^6^ CAR-T cells/kg) (Supplementary Fig. 4 and Supplementary Table 2). The baseline demographics and clinical characteristics of these 15 infused patients are listed in Table 1. The median age was 59 years (range, 35–70); six patients had pancreatic cancer, three had biliary tract cancer, and the other six had gastric cancer, tubal cancer, esophageal cancer, ovarian cancer, cervical cancer, and triple-negative breast cancer. All 15 infused patients had experienced treatment failure with one or more conventional chemotherapy regimens (Supplementary Table 3) and presented refractory or recurrent metastatic disease mainly involving the lymph nodes and liver at baseline. Twelve of the 15 patients had an ECOG performance status of 1–2, and three had an ECOG performance status of 3. MSLN positivity was confirmed by immunohistochemistry (Supplementary Table 4 and Supplementary Fig. 5). PD-L1 expression at baseline was assessed in 15/17 patients enrolled (14/15 patients infused) with available samples (Supplementary Table 5). Five patients had an expression of PD-L1 on ≥1% of tumor cells, which was the cutoff used in this study to determine PD-L1 immunohistochemistry positivity.

**Table 1:** Baseline demographics and clinical characteristics of the treated patients

Characteristics	No. (15)	%
*Age, years*		
Median	59	
Range	35–70	
*Sex*		
Male	8	53.3
Female	7	46.7
*ECOG performance status*		
1	8	53.3
2	4	26.7
3	3	20.0
*Pathology*		
Pancreatic cancer	6	40.0
Biliary tract cancer	3	20.0
Gastric cancer	1	6.7
Tubal cancer	1	6.7
Esophageal cancer	1	6.7
Ovarian cancer	1	6.7
Cervical cancer	1	6.7
Triple-negative breast cancer	1	6.7
*Number of prior anticancer therapies*		
Median	10	
Range	1–31	

*ECOG* Eastern Cooperative Oncology Group

### Characterization of clinical-grade MPTK-CAR-T cell products

**Fig. 2:**
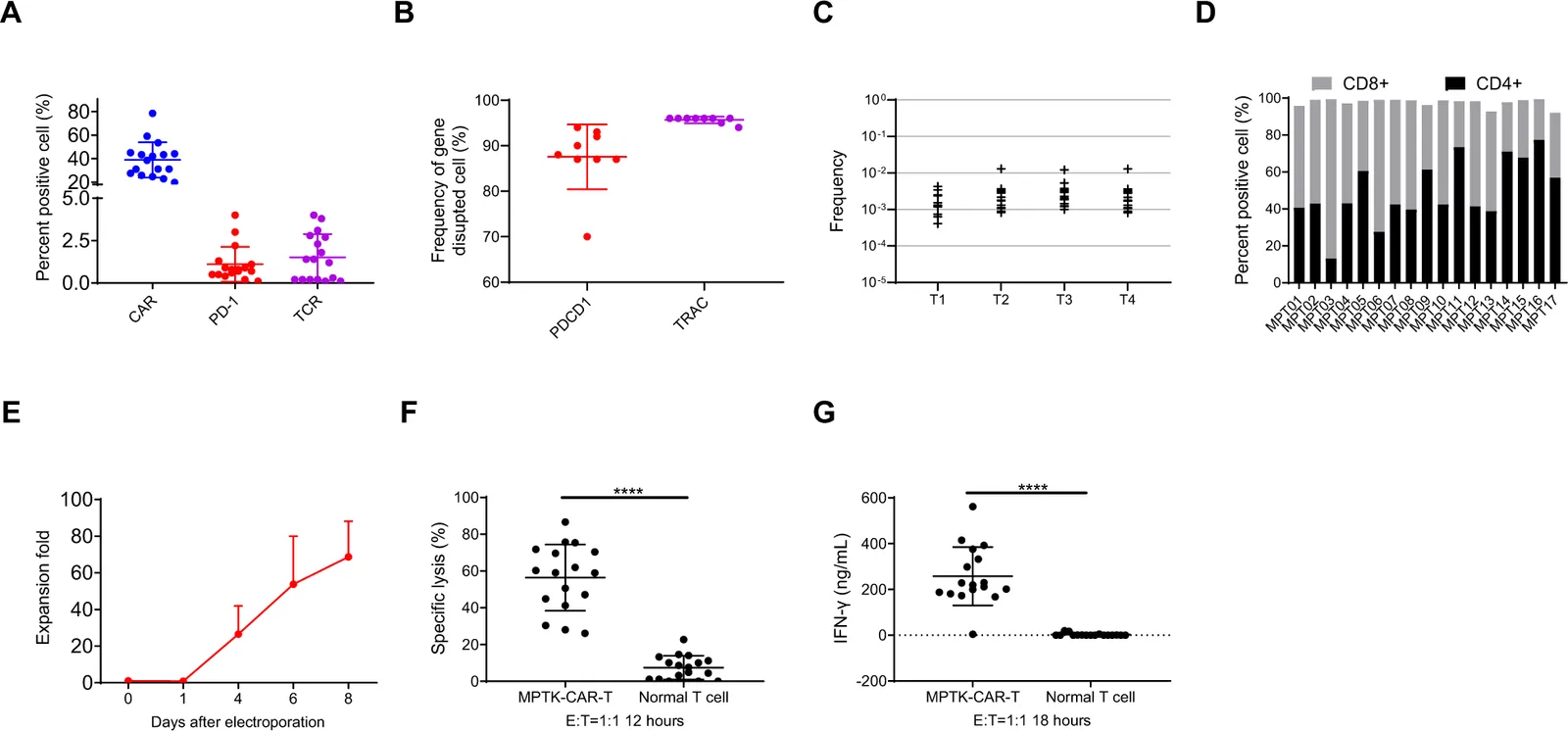
Characterization of clinical-grade MPTK-CAR-T cell products. **A** Flow cytometric analysis of CAR, PD-1, and TCR surface expression in MPTK-CAR-T cell products. Individual data points and the mean with SD are shown. **B** The frequencies of gene editing for *PDCD1* and *TRAC* in 9 MPTK-CAR-T infusion products. Individual data points and the mean with SD are shown. **C** Translocation frequency in 15 MPTK-CAR-T cell products. T1 to T4 represent four different possible translocation events. **D** CD8/CD4 ratio of MPTK-CAR-T cell products. **E** In vitro expansion of MPTK-CAR-T cell products. The fold changes in the cell number of MPTK-CAR-T cells after electroporation are shown. **F** Cytotoxicity assay using MPTK-CAR-T cells and normal T cells as effectors and the CRL5826-PD-L1 cell line as a target. Individual data points and the mean with SD at an E:T ratio of 1:1 are shown. MPTK-CAR-T cells vs. normal T cells, ****P < 0.0001. **G** IFN-γ release assay using MPTK-CAR-T cells and normal T cells as effectors and the CRL5826-PD-L1 cell line as a target. Individual data points and the mean with SD at an E:T ratio of 1:1 are shown. MPTK-CAR-T cells vs. normal T cells, ****P < 0.0001. Statistics were analyzed with an unpaired *t* test (**F** and **G**)

MPTK-CAR-T cells were successfully generated for all 17 enrolled patients. The mean CAR transduction efficiency was 38.9% (range, 19.7–78.5%; Fig. 2A), the mean PD-1 surface expression was 1.1% (range, 0.1–4.0%; Fig. 2A and Supplementary Fig. 6, Supplementary Table 6), and the mean TCR surface expression was 1.5% (range, 0.1–4.0%; Fig. 2A and Supplementary Fig. 6, Supplementary Table 6); we assessed the editing efficiency at the DNA level in 9/17 MPTK-CAR-T cell products, and the mean frequencies of gene editing for *PDCD1* and *TRAC* were 87.6% (range, 70.0–94.0%) and 95.7% (range, 94.0–96.0%), respectively (Fig. 2B).

Simultaneous introduction of multiple DNA double-strand breaks could lead to the generation of chromosomal translocations. To evaluate the probability of such events, we quantified the translocation frequencies of all four possible events that could be derived from the simultaneous editing of *TRAC* on chromosome 14 and *PDCD1* on chromosome 2 using qPCR. The translocation frequencies ranged from 6 × 10^−4^ to 2 × 10^−2^ (Fig. 2C), a range that was similar to that of dual-gene editing with transcription activator-like effector nuclease (TALEN) in preclinical and clinical studies and tri-gene editing with CRISPR-Cas9 in a clinical study [21–23].

MPTK-CAR-T cell products contained a mean of 49.4% CD4+ (range, 13.1– 77.4%) and a mean of 48.2% CD8+ (range, 22.0–86.3%) T cells (Fig. 2D), and they had expanded 68-fold on average (range, 36–102) at 6–8 days after electroporation (Fig. 2E and Supplementary Fig. 7). MPTK-CAR-T cells had substantial cytotoxic effects against the cell line CRL5826-PD-L1 and released substantial amounts of IFN-γ (Fig. 2F and G).

### Safety

Fifteen patients received a total of 25 infusions, with 8 patients receiving multiple infusions (supplementary Table 2). We observed no evidence of neurotoxicity or CRS in any of the 15 subjects. No indications of autoimmune reaction were found in any of the 15 patients. A panel of 16 serum cytokines was examined at specified time points after MPTK-CAR-T cell infusion, and five of the 15 patients had >10-fold elevations in either IL-6 (MPT05 and MPT 16), IL-8 (MPT17), or IL-10 (MPT13 and MPT14) after infusion, but no other cytokines were elevated over 10-fold from baseline in any of the 15 subjects (Supplementary Fig. 8).

There was no overt clinical evidence of pleuropericarditis or peritonitis. Two patients (MPT07 and MPT16) developed new-onset grade<3 pleural, pericardial or peritoneal effusion, which did not further deteriorate after subsequent infusion in either patient; four patients (MPT01, MPT02, MPT08, and MPT13) with pleural, pericardial, or peritoneal cavity effusions at baseline exhibited increased effusions after CAR-T cell infusion (Table 2 and Supplementary Fig. 9) that were manageable with catheter drainage. Patients MPT01 (grade 1 ascites and pleural effusion preinfusion) and MPT02 (grade 3 ascites preinfusion) mainly exhibited an increase in ascites within 2 weeks after infusion. Patient MPT08 with mild pericardial effusion, pleural effusion and ascites at baseline experienced a mild increase in pleural and pericardial effusion within 1 month of infusion, but noteworthy increases in pleural and pericardial effusion were detected by computed tomography (CT) performed at day 56 after infusion. For patient MPT13 with mild pleural effusion and pericardial effusion preinfusion, CT imaging at day 12 and day 34 after infusion showed a mild and notable increase in pleural effusion compared to that at baseline.

**Table 2:** Pleural, pericardial or peritoneal cavity effusions suspected to be related to MPTK-CAR-T cell infusion

Type of event	≤4 Wk After infusion (*N* = 15)		>4 Wk After infusion (*N* = 15)	
	Grade < 3	Grade ≥ 3	Grade < 3	Grade ≥ 3
	Number of patients (Percent)			
Pleural effusion	3 (20.0)	−	1 (6.7)	2 (13.3)
Pericardial effusion	1 (6.7)	−	1 (6.7)	1 (6.7)
Ascites	1 (6.7)	2 (13.3)	−	−

Moreover, T cells in PB specimens taken from 1 to 7 months after MPTK-CAR-T cell infusion were acquired from four patients (MPT07, day 208; MPT09, day 198; MPT14, day 145; and MPT17, day 37), and the TCR β-chain region variability was analyzed by flow cytometry. No profound skewing was observed in CD4+ or CD8+ T cells from any of the four patients (the result of patient MPT07 is represented in Supplementary Fig. 10), suggesting no oligoclonal/monoclonal pattern of the circulating T cells after MPTK-CAR-T cell infusion. This result indicated that gene editing did not lead to abnormal amplification of rare T cell clones.

### Detection of MPTK-CAR-T cells in vivo

**Fig. 3:**
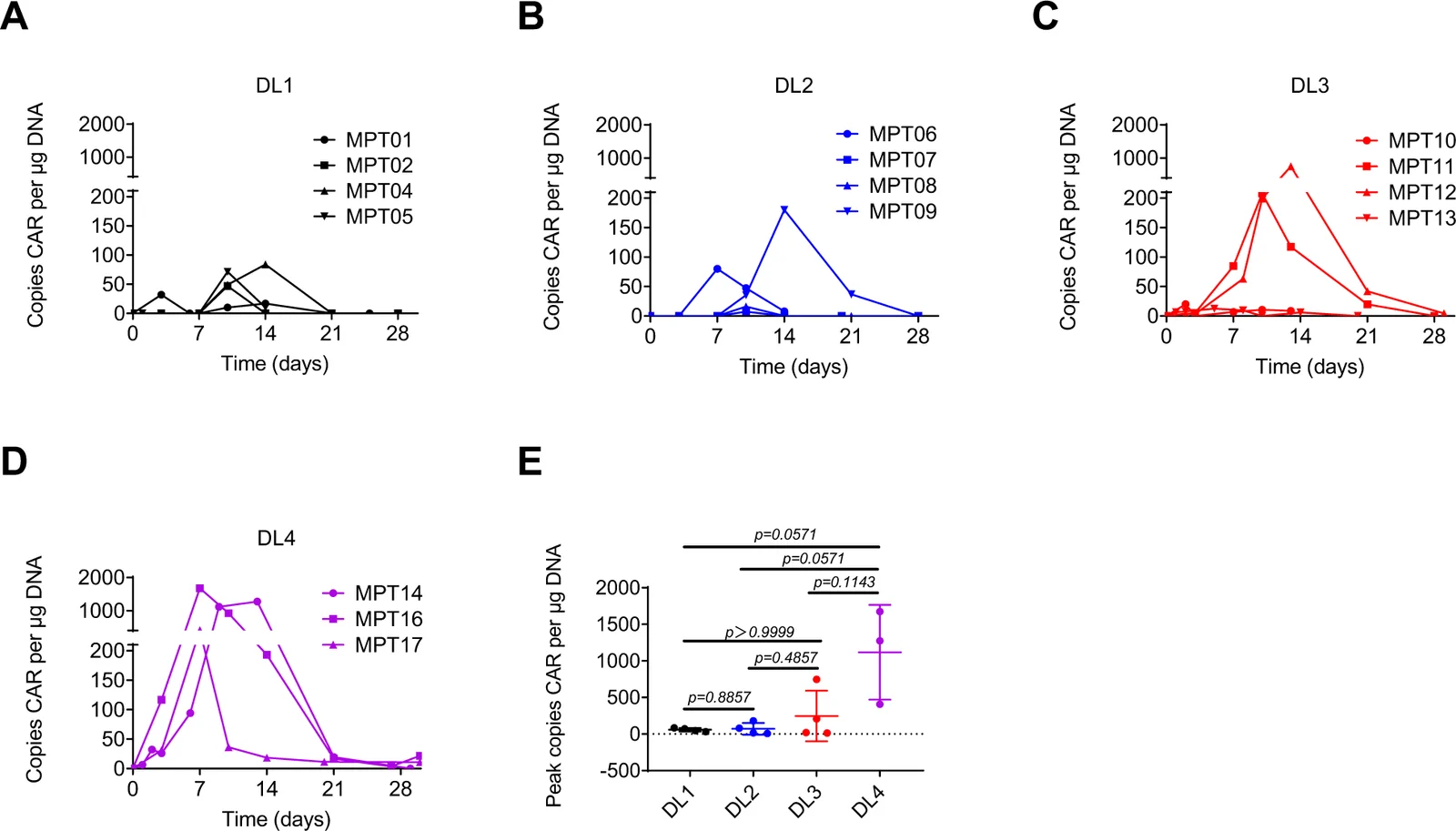
Cellular kinetics of MPTK-CAR-T cells after the first infusion. **A–D** In vivo expansion and persistence of MPTK-CAR-T cells as measured by qPCR in peripheral blood at each dose level. DL dose level. **E** Comparison of the mean peak levels of MPTK-CAR-T cells in peripheral blood at each dose level. Statistical significance was determined by the Mann–Whitney test

We detected MPTK-CAR-T cells in PB by qPCR after infusion. All patients had detectable circulating MPTK-CAR-T cells, which peaked within 7–14 days after infusion and became undetectable beyond one month (Fig. 3A–D). Higher levels of MPTK-CAR-T cells were detected in patients treated with DL4 or DL3, while the differences in mean peak levels between any two groups were not statistically significant (Fig. 3E).

Of the eight patients who received a repeat infusion of MPTK-CAR-T cells at an equivalent or higher dose, seven patients failed to achieve engraftment after the second infusion, although the expansion of MPTK-CAR-T cells was noted after the first infusion in these patients (Supplementary Fig. 11). Among the aforementioned four patients with worsened effusions after infusion (MPT01, MPT02, MPT08, and MPT13), MPTK-CAR-T cells were detected by qPCR in ascites from MPT01 and MPT02 and in pericardial effusion from MPT08 but were undetectable in pleural effusion samples from MPT13. Moreover, MPTK-CAR-T cells expanded with a higher copy number of the CAR gene in effusions than in PB at each time point after infusion but became undetectable beyond day 70 (Supplementary Fig. 12).

### MPTK-CAR-T cells traffic to tumor sites

**Fig. 4:**
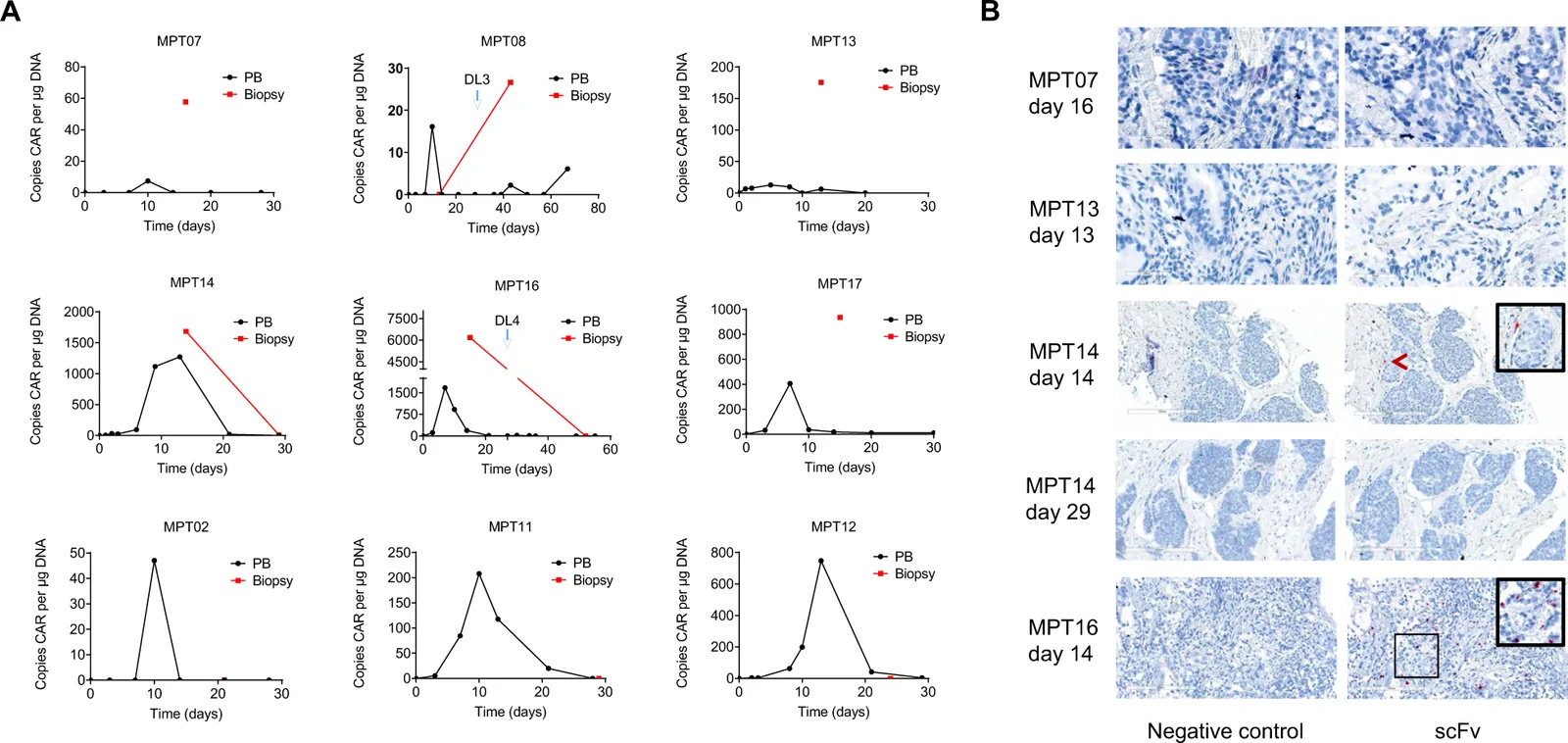
Trafficking and kinetics of MPTK-CAR-T cells in tumor samples. **A** Determination of MPTK-CAR-T cells by qPCR in tumor biopsy samples at 2–4 weeks after infusion (blue arrowheads indicate the second infusion). DL dose level, PB peripheral blood. **B** Determination of MPTK-CAR-T cells in tumor biopsy samples by RNAscope ISH specifically for the scFv of the P4 CAR. RNAscope ISH, RNAscope in situ hybridization

Nine subjects had a biopsy of tumor metastasis (4 liver, 4 lymph node, and 1 chest wall metastases) at 2–4 weeks after MPTK-CAR-T cell infusion to explore the trafficking and persistence of MPTK-CAR-T cells in tumors, as assessed by qPCR and RNAscope in situ hybridization (RNAscope ISH). In almost all tumor biopsy samples taken at 2 weeks after infusion (MPT07, MPT08, MPT13, MPT14, MPT16, and MPT17), MPTK-CAR-T cells were detected, at a particularly high level in MPT14 and MPT16, implying effective trafficking of these cells to tumors (Fig. 4A). With respect to the tumor biopsy samples taken at 3–4 weeks after infusion (taken from MPT02, MPT11, and MPT12), no qPCR signal of MPTK-CAR-T cells was detected even though signals were detected in PB samples from these patients. In addition, MPTK-CAR-T cells in the chest wall metastasis biopsy samples from MPT14 were detected at day 14 but became almost undetectable at day 29. These observations suggest that MPTK-CAR-T cells could traffic and infiltrate into tumors but could not persist beyond one month in tumors, similar to the observations in PB samples (Fig. 4A).

In addition to qPCR assays, five tumor biopsy samples (from four patients, MPT07, MPT13, MPT14, and MPT16) obtained at the same time point were examined by RNAscope ISH. Positive RNAscope ISH signals of MPTK-CAR-T cells were identified in tumor samples from MPT14 and MPT16 (biopsied 2 weeks after infusion), in which a high copy number of the CAR gene was also detected by qPCR (Fig. 4B). In contrast, RNAscope ISH failed to detect MPTK-CAR-T cell signals in the other tumor samples, which had a lower copy number of the CAR gene (Fig. 4B), suggesting a higher detection sensitivity of qPCR than RNAscope ISH.

### Clinical responses and outcomes after MPTK-CAR-T cell infusion

**Fig. 5:**
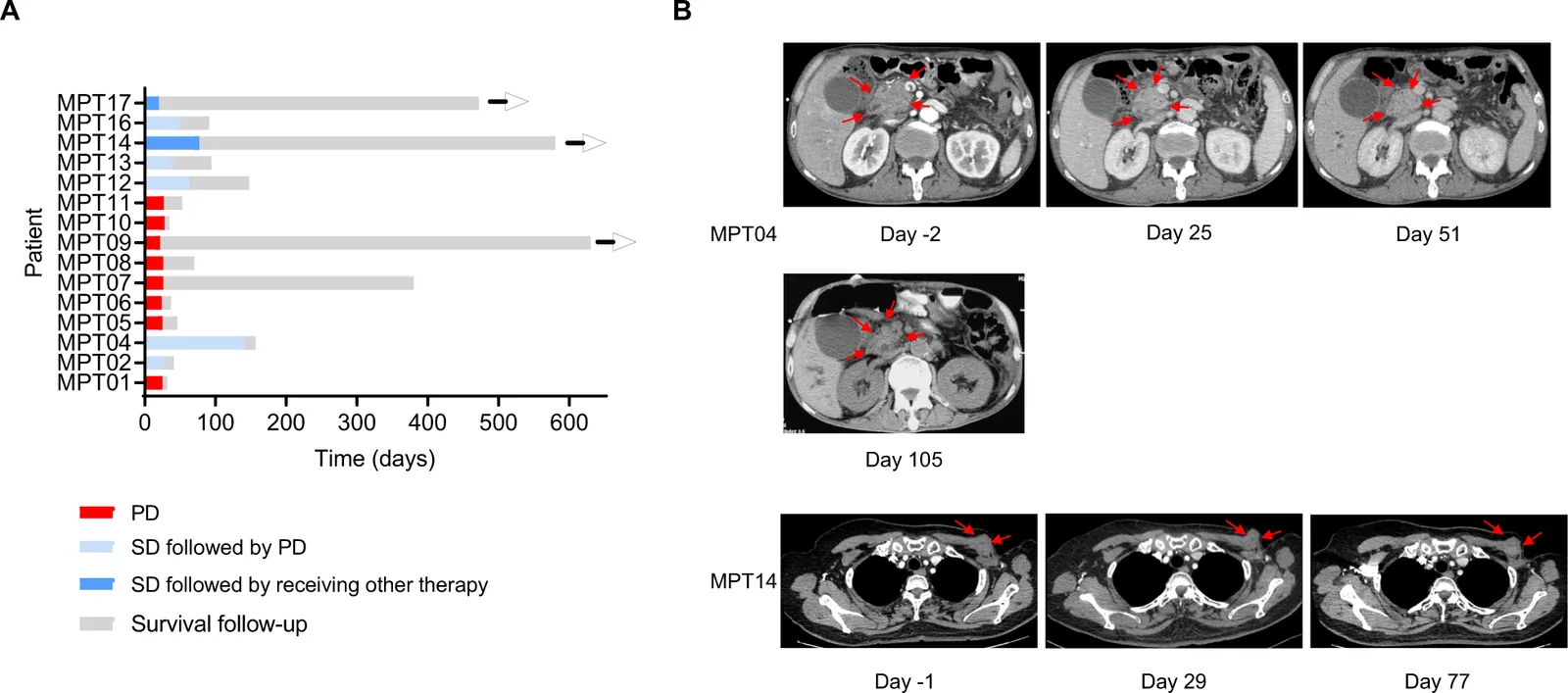
Clinical outcomes after MPTK-CAR-T cell infusion. **A** Swimmer plot indicating the disease status and survival of all 15 treated patients (black arrowheads indicate ongoing survival). PD progressive disease and SD stable disease. **B** Imaging changes in the target lesion before and at several time points after infusion

Of the 15 patients, 2 (13%) achieved a BOR of SD per the Response Evaluation Criteria in Solid Tumors (RECIST) 1.1. Seven of the 15 patients achieved SD 3–4 weeks after infusion, but the response was maintained only in 2 out of 7 patients at a follow-up duration of 8–12 weeks. The median PFS of those 7 patients with SD was 7.1 weeks (range, 2.9–20.1) (Fig. 5A and Supplementary Table 2). Patient MPT04, a 61-year-old man who had gastric cancer with lymph node metastasis, received a total of three MPTK-CAR-T cell infusions (1 DL1,1 DL2, and 1 DL3); he obtained SD with up to 18% tumor shrinkage by day 51 that was maintained at day 105 (Fig. 5B) and survived for 5.2 months after the first infusion. Patient MPT14, a 55-year-old woman who had a recurrence of a triple-negative breast cancer chest wall metastatic lesion in situ after surgical excision and radiotherapy, received 2 doses of MPTK-CAR-T cell infusions at DL4 and achieved SD with up to 20% tumor shrinkage at day 77 after the first infusion (Fig. 5B). She then received another investigational product while maintaining a stable disease. Eight patients had progressive disease, and the majority of them died within 2 months after infusion (Fig. 5A and supplementary Table 2). As of April 29, 2020, the median overall survival was 3.0 months (range, 1.1–21.0) and 4.9 months (range, 1.4–19.3) for all 15 patients and the 7 patients with SD at weeks 3–4 after infusion, respectively.

Moreover, serologic tumor markers were measured pre-and post-MPTK-CAR-T cell infusion for all 15 patients to evaluate the impact of treatment. Among the 11 patients with abnormal baseline CA19-9 levels, several showed a decrease in CA19-9 levels of up to 57.5% after infusion, suggesting the antitumor activity of MPTK-CAR-T cells in these patients. However, the effect on CA19-9 levels was not maintained, which is consistent with the lack of persistence of MPTK-CAR-T cells in vivo (Supplementary Fig 13).

### Evaluating the importance of the TCR for the persistence of MPTK-CAR-T cells in vivo

**Fig. 6:**
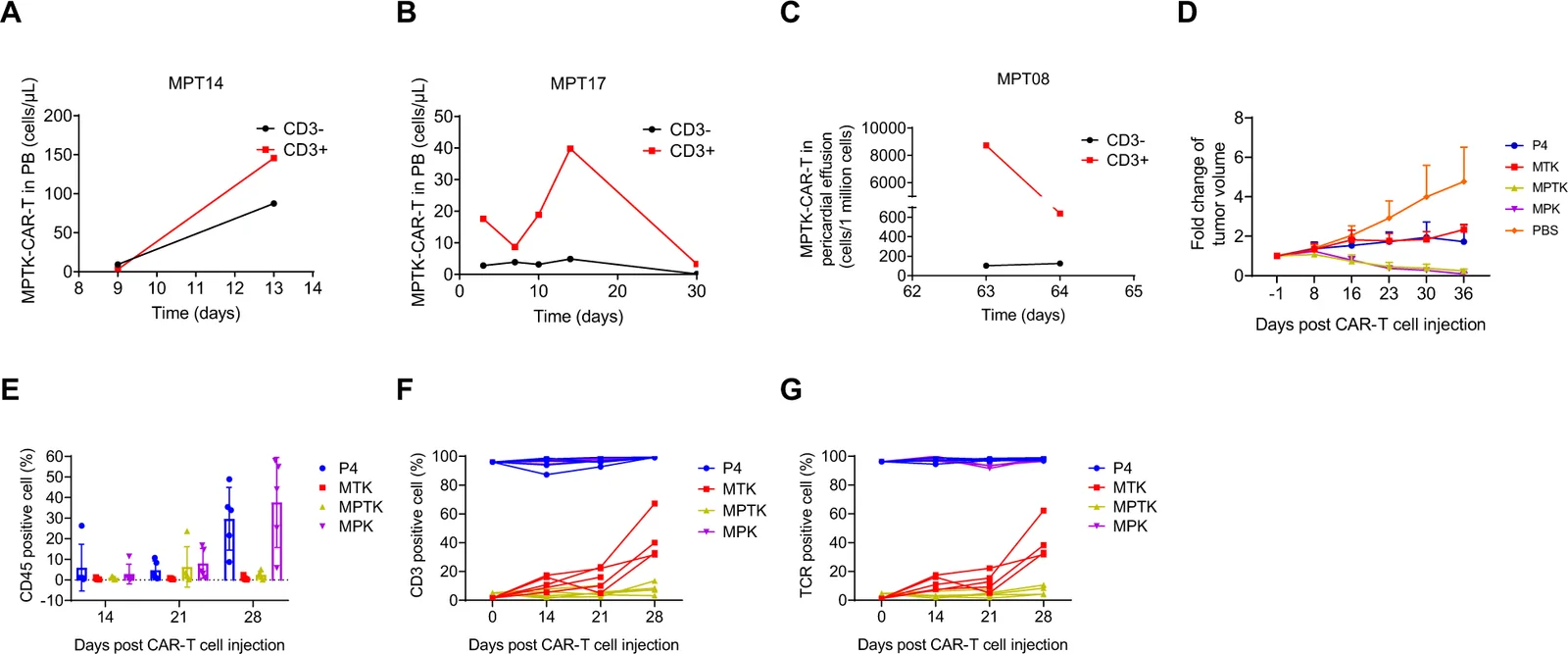
Cellular kinetics of MPTK- and MTK-CAR-T cells in vivo. **A–C** In vivo expansion and persistence of MPTK-CAR-T cells in peripheral blood or pericardial effusion from three patients. **D–G** The expansion and persistence of MPTK-CAR-T cells in CRL5826-PD-L1 tumor-bearing NPG mice. Tumor growth (**D**), and PB analysis of the proportions of human CD45-positive cells (**E**), human CD3-positive cells (**F**) and human TCR-positive cells (**G**) after CAR-T cell intravenous injection (*n* = 5 animals per group)

To identify the possible cause of the poor persistence of MPTK-CAR-T cells in vivo, we further analyzed PB samples from patients MPT14 and MPT17, the only two patients in whom MPTK-CAR-T cells could be reliably detected by flow cytometry analysis. Surprisingly, we found that CD3+ CAR-T cells (CD3 is strictly correlated with the TCR signal by flow cytometry analysis, Supplementary Fig. 2B) accounted for the majority of cells (Fig. 6A and B, Supplementary Fig. 14A), while <5% of the MPTK-CAR-T cells in the products preinfusion were TCR+ and CD3+ cells (Fig. 2A and Supplementary Fig. 6, Supplementary Table 6). We further examined the subpopulations of circulating CAR+ T cells taken from patient MPT14 at day 13 and found that PD-1-negative CAR+ T cells rather than PD-1-positive CAR+ T cells were the majority in both the TCR-positive CAR-T cell and TCR-negative CAR-T cell compartments (Supplementary Fig. 14B). In addition, we identified a high percentage of CD3-positive CAR-T cells in the pericardial effusion from MPT08 (Fig. 6C and Supplementary Fig. 15). These results suggest that disruption of the TCR in MPTK-CAR-T cells leads to disadvantages in proliferation and/or engraftment in vivo.

To further validate the clinical observation that suggested the involvement of the TCR in CAR-T cell persistence in vivo, we performed a more detailed analysis. We generated P4 CAR-T cells with *TRAC* single knockout (MTK) and MPTK-CAR-T cells from the same donor and evaluated their proliferation capability both in vitro and in vivo. Consistent with our previous data, these three types of cells had similar in vitro proliferation capability both in the presence and absence of tumor antigen stimulation (Supplementary Fig. 16A–C). When these cells were challenged by multiple rounds of addition of fresh tumor cells, the CAR-T cell numbers in different samples were very similar in each round (Supplementary Fig. 16D).

Next, we injected 2 × 10^7^ CAR-T cells intravenously into the CRL5826-PD-L1 CDX model and obtained data consistent with our previous preclinical animal data (Fig. 1F and J), as the P4 and MTK-CAR-T cells inhibited tumor growth compared with the PBS control group, while MPK- and MPTK-CAR-T cell infusion almost cleared the tumor by day 36 (Fig. 6D). To monitor the in vivo persistence of CAR-T cells in greater detail, we started to collect PB samples from each mouse at 14 days after injection and weekly afterward. While the percentage of human CD45-positive cells in all mouse PB samples was similar at day 14, we found that by day 28, the percentages of P4 and MTK-CAR-T cells were significantly higher than those of MTK- and MPTK-CAR-T cells (Fig. 6E). Furthermore, within the MTK-CAR-T cells, which had <1% TCR+ cells in preinfusion samples, the percentage of TCR- and CD3-positive cells increased over time, reaching more than 30% by day 28 (Fig. 6F). A similar trend of an increase in TCR+ cells was also observed in MPTK-CAR-T cell samples (Fig. 6G). These results were similar to our observations in clinical samples, further indicating that the TCR-negative CD3− CAR-T cells had proliferation and/or engraftment disadvantages in vivo.

## Discussion

In this first-in-human, proof of concept, dose-escalation study, we explored the feasibility, safety, and cellular kinetics of MPTK-CAR-T cells in 15 patients with metastatic solid tumors. We found no overt evidence of autoimmune reaction, on-target/off-tumor toxicities such as pleuropericarditis or peritonitis, or unexpected toxicities after administration of MPTK-CAR-T cells at a dose of up to 1.1 × 10^7^/kg, and no aberrant T cell clones arose up to 7 months after infusion. MPTK-CAR-T cells could not persist beyond 6 weeks after the first infusion in PB, at the tumor site, or in effusion samples in almost all patients. Failure to achieve engraftment of MPTK-CAR-T cells after repeat infusions were observed, and we suspect this was due to the anti-CAR humoral or cellular immune response, as reported previously [2, 3, 24, 25].

We note that while preliminary safety was suggested by this study, the long-term safety of CRISPR-engineered T cells remains unknown due to the short-term persistence of MPTK-CAR-T cells in vivo. Stadtmauer et al. [23] recently reported in their clinical study of CRISPR-engineered TCR-T cells with PD-1 and natural TCR disruption that chromosomal translocations decreased over time, and no evidence of overproliferation was observed up to 170 days after infusion in one patient.

The effusion increase observed in four patients (MPT01, MPT02, MPT08, and MPT13) could be due to the following factors: (1) progressive disease at the site of effusion; (2) MPTK-CAR-T cell-mediated effects on tumor cells at the site of effusion; and (3) MPTK-CAR-T cell-mediated on-target/off-tumor effects on normal serosal cells of the pleura, peritoneum, and pericardium. In the case of those patients with ascites (MPT01 and MPT02) and/or pleural effusion (MPT01 and MPT13) in parallel with existing tumors in the peritoneal cavity (MPT01 and MPT02) or probable existing tumors on the left pleural surfaces (MPT13) at baseline, it is difficult to delineate the real role of those three factors in increased ascites or pleural effusion, which may be a consequence of the synergy of multiple factors. For patient MPT08, we could not exclude that the MPTK-CAR-T cells mediated the on-target/off-tumor effects on normal serosal cells of the pericardium that led to the increased pericardial effusion given that the peak level of expanded MPTK-CAR-T cells (11,889 copies per μg DNA) in the drained pericardial effusion at days 63–67. Although an increased risk of worsened effusion after MPTK-CAR-T cell infusion was seen in those patients, the effusion was generally manageable.

In patients, disruption of PD-1 in MSLN-directed CAR-T cells did not induce antitumor activity as notably as combining MSLN-targeted CAR-T cell therapy with an anti-PD-1 agent [7], which may be because the additional TCR disruption in our study likely resulted in poor expansion and lack of long-term persistence of MPTK-CAR-T cells in vivo. In addition, CAR-T cells were administered via I.V. injection in our study, while Adusumilli et al. injected CAR-T cells intrapleurally [7]. The different delivery routes may also contribute to the difference in clinical response. Anti-PD-1 antibodies mainly work by blocking the interaction between PD-1 on T cells and PD-L1 on tumor cells. Paucity or low expression of PD-L1 on tumor cells is a well-defined factor associated with primary resistance to anti-PD-1 antibody treatment [26, 27], while high expression of PD-L1 commonly indicates a better clinical response [28]. Therefore, the low rate of PD-L1 positivity (36% with a 1% cutoff value and 21% with a 5% cutoff value) in patients may have also contributed to the limited clinical response in our study. Of note, in contrast to knocking out PD-1 in CAR-T cells, combining CAR-T cell therapy with anti-PD-1 antibody therapy could restore the activity of not only the infused CAR-T cells but also other innate immune cells, including tumor-infiltrating lymphocytes, in the tumor microenvironment and may provide more effects to eradicate cancer cells. To some extent, our poor clinical response implies that CAR-T cell-intrinsic modification alone, for example, knocking out PD-1 in CAR-T cells, may not be enough to induce promising outcomes in the treatment of patients with solid tumors, highlighting that more endeavors are needed to explore cell-intrinsic and cell-extrinsic combinational therapy approaches to affect infused CAR-T cells, the innate immune system, and the tumor milieu.

Expression of a CAR endows CAR-T cells with dual specificity via the CAR and the endogenous TCR. Although CAR-T cell therapy has been successful in treating B cell malignancies, the effects of endogenous TCR signaling in CAR-T cell biology have not been well defined. Cumulative data from preclinical and clinical studies suggest that endogenous TCR signaling is not required to obtain fully functional CAR-T cells [29–31], while it could negatively affect CAR-T cell expansion and functionality [32, 33].

In contrast, we unexpectedly observed that TCR-positive T cells, accounting for <5% of the infused cell product, became the main fraction after infusion in three patients, suggesting that the TCR-negative CAR-T cells had inferior expansion and/or persistence in patients compared to the TCR-positive CAR-T cells. We further confirmed this observation in mouse models. Currently, it is well known that T lymphocytes exhibit low-level, constitutive signaling in the basal state (tonic signaling), which is triggered by transient interactions between the TCR and self-peptide–MHC complexes in lymphoid organs [34]. There is growing evidence suggesting that endogenous TCR tonic signaling is important for T-cell differentiation and effector function [35]. In our study, endogenous TCR tonic signaling was likely to play a beneficial role in promoting the persistence of CAR-T cells. It must be emphasized that the effect of the natural TCR on CAR-T cell persistence in vivo was observed in the scenario of low-level engraftment, while whether this holds true in the case of higher CAR-T cell engraftment, such as the engraftment seen for combination strategies including lymphodepleting chemotherapy, remains to be seen.

Recently, Stadtmauer et al. [23] reported high-level engraftment and long-term persistence of infused TCR-T cells with natural TCR and PD-1 disruption induced by CRISPR-Cas9 technology in three patients with either myeloma or sarcoma. In this case, a synthetic TCR replaced the endogenous TCR, and the lymphodepleting chemotherapy might have provided a better environment for TCR-T cells to engraft. During the preparation of our manuscript, a study was published reporting the observation of reduced CAR-T cell persistence in animal models when CD19 CAR-T cells were used to treat blood tumors [36]. This report is consistent with our observations from using CAR-T cells to treat solid tumors in animal models. More importantly, our clinical study also found reduced persistence of TCR-deficient CAR-T cells in patients. Taken together, these data imply a critical role of natural TCRs in the proliferation capacity of CAR-T cells in vivo. Interestingly, CAR-T cells lacking a TCR had the same proliferation capability in vitro, both with and without tumor antigen encounter (Supplementary Fig. 16). The crosstalk between endogenous TCR and CAR signaling needs to be studied in greater detail and might be the key to improving CAR-T cell efficacy in the treatment of solid tumors, and our in vitro and in vivo preclinical models provide good systems for this purpose.

In conclusion, we demonstrated that CRISPR-Cas9-engineered CAR-T cells with PD-1 disruption did not elicit unconstrained proliferation or persistence in humans and did not induce unexpected safety issues, while the effects of removing the endogenous TCRs from CAR-T cells in the treatment of solid tumors should be further addressed.

Supplementary information is available at Cellular & Molecular Immunology’s website.

## Supplementary information

Supplementary information file

Supplementary figure
