# Supplementary material for: Phase I study of CAR-T cells with PD-1 and TCR disruption in mesothelin-positive solid tumors
Source: Cell Mol Immunol. 2021 Aug 11;18(9):2188–98. doi: 10.1038/s41423-021-00749-x (PMC8429583; doi:10.1038/s41423-021-00749-x)
Supplement: Supplementary file 1 — Supplementary information file [file 41423_2021_749_MOESM1_ESM.pdf]

Supplementary information file

Phase I study of CAR-T cells with PD-1 and TCR disruption in mesothelin-positive solid tumors

Zhenguang Wang[1, 2], Na Li[3], Kaichao Feng[2], Meixia Chen[2], Yan Zhang[2], Yang Liu[2], Qingming Yang[2], Jing Nie[2], Na Tang[3], Xingying Zhang[3], Chen Cheng[3,4], Lianjun Shen[5], Jiaping He[5], Xun Ye[5], Wei Cao[5], Haoyi Wang[3,6], Weidong Han[1,2]

[1] Medical School of Chinese PLA, No. 28 Fuxing Road, Beijing, 100853, China.

[2] Department of bio-therapeutic, the first Medical Center, Chinese PLA General Hospital, No. 28 Fuxing Road, Beijing, 100853, China.

[3] State Key Laboratory of Stem Cell and Reproductive Biology, Institute of Zoology, Chinese Academy of Sciences, 1 Beichen West Road, Beijing, 100101, China.

[4] School of Life Sciences, University of Science and Technology of China, No.96 Jinzhai Road, Hefei, 230026, China.

[5] Gracell Biotechnologies (Shanghai) Co., Ltd, 926 Yishan Road, Shanghai, 200233, China.

[6] Institute for Stem Cell and Regeneration, Chinese Academy of Sciences, 1 Beichen West Road, Beijing, 100101, China.

These authors contributed equally to this work: Zhenguang Wang, Na Li, Kaichao Feng

Address correspondence to: Weidong Han, Medical School of Chinese PLA, No. 28 Fuxing Road, Beijing, 100853, China. Phone: 86-10-66937463, Email: hanwdrsw69@yahoo.com or Haoyi Wang, State Key Laboratory of Stem Cell and Reproductive Biology, Institute of Zoology, Chinese Academy of Sciences, 1 Beichen

West Road, Beijing, 100101, China. Phone: 86-10-82619213, Email: wanghaoyi@ioz.ac.cn or Wei Cao, Gracell Biotechnologies (Shanghai) Co., Ltd, 926 Yishan Road, Shanghai, 200233, China. Phone: 86-21-64031375, Email: william.cao@gracellbio.com.

This file includes:

Supplementary Figure 1. *PDCD1* knock-out P4 CAR-T cells exhibited superior anti-tumor efficiency *in vitro.*

Supplementary Figure 2. Characterization of *PDCD1/TRAC* double knock-out P4 CAR-T cells.

Supplementary Figure 3. *PDCD1* knock-out P4 CAR-T cells exhibited superior anti-tumor efficiency *in vivo*.

Supplementary Figure 4. Patient recruitment and screening.

Supplementary Figure 5. Detection of MSLN expression by Immunohistochemistry.

Supplementary Figure 6. Representative PD-1 and TCR surface expression in MPTK-CAR-T cell infusion product.

Supplementary Figure 7. *In vitro* expansion of MPTK-CAR-T cell products for all 17 patients enrolled.

Supplementary Figure 8. Serum cytokine changes after MPTK-CAR-T cell infusion.

Supplementary Figure 9. Four patients with baseline serous cavity effusions experienced increased effusions after MPTK-CAR-T cell infusion.

Supplementary Figure 10. Flow cytometric analysis of TCR Vβ family usage after

MPTK-CAR-T cell infusion.

Supplementary Figure 11. Cellular kinetics of MPTK-CAR-T cells in peripheral blood after repeat infusions.

Supplementary Figure 12. Cellular kinetics of MPTK-CAR-T cells in ascites or pericardial effusion after infusion.

Supplementary Figure 13. CA19-9 levels after MPTK-CAR-T cell infusion.

Supplementary Figure 14. Representative flow cytometry plots of circulating MPTK-CAR-T cells after infusion.

Supplementary Figure 15. Flow cytometric evaluation of MPTK-CAR-T cells in pericardial effusion after infusion.

Supplementary Figure 16. Proliferation of *TRAC* knock-out P4 CAR-T cells when encountering antigen *in vitro.*

Supplementary Table 1. Off-target candidates genotyping.

Supplementary Table 2. Clinical outcome after MPTK-CAR-T cell infusion.

Supplementary Table 3. Treatment history of all 15 patients infused.

Supplementary Table 4. Results of MSLN Immunohistochemistry.

Supplementary Table 5. Results of PD-L1 Immunohistochemistry.

Supplementary Table 6. PD-1 and TCR expression in MPTK-CAR-T cell products and control cells.

Supplementary Figures

Supplementary Figure Legends

Supplementary Figure 1. *PDCD1* knock-out P4 CAR-T cells exhibited superior anti-

tumor efficiency *in vitro.*

A Schematic diagram of *PDCD1* and *TRAC* target region. Red indicates the sgRNA-targeting site. **B** Schematic of the anti-mesothelin CAR-T cells with CD28-CD3ζ signaling domain. **C** Mesothelin and PD-L1 expression on CRL5826 and CRL5826-PD-L1 cells by flow cytometry. **D** *PDCD1* gene editing efficiency in MPK-CAR-T cells measured by inference of CRISPR edits (ICE) assay. F and R represent two different direction of Sanger sequencing used for ICE. **E** Cytotoxicity of P4 and MPK CAR-T cells cocultured with CRL5826-PD-L1 at lower E:T ratios for 4 days. The assays in D and E were repeated in two donors and similar results were obtained. Statistics was performed by ANOVA with Tukey's multiple-comparisons test in E. Data were represented as mean ± standard deviation (SD). *P < 0.05; ****P < 0.0001.

Supplementary Figure 2. Characterization of *PDCD1*/*TRAC* double knock-out P4 CAR-T cells.

A *PDCD1* and *TRAC* gene editing efficiencies in MPTK-CAR-T cells measured by ICE assay. **B** Expression of CD3 and TCR of P4 and MPTK CAR-T cells by flow cytometry. **C-F**, Basic characteristics. Transduction efficiency **C**, proliferation **D,** CD4/8 ratio **E**, and T cell subsets **F** of MPTK, P4 CAR-T and T cells, analyzed by flow cytometry. The assays were repeated using cell from two donors and similar results were obtained.

Supplementary Figure 3. *PDCD1* knock-out P4 cells exhibited superior anti-tumor efficiency *in vivo.*

A Mesothelin expression in two PDX models**. B** Scheme of *in vivo* assay to test the anti-tumor function of CAR-T cells in C-D. NPG mice were subcutaneously injected with

CRL5826-PD-L1 cells (CDX model). When tumor grew to about 200-300 mm^3, MPK, P4 CAR-T cells or PBS were injected intratumorally twice separated by seven days. Bioluminescence imaging, weight or tumor volume of mice were measured weekly. **C-D,** the anti-tumor function of MPK-CAR-T cells derived from two donors in CDX model. Fold changes of tumor bioluminescence signal were measured over time after intra-tumor administration of CAR-T cells. Two independent experiments each using CAR-T cells derived from independent donors were shown in C and D (n ≥ 4 animals per group). **E** Scheme of *in vivo* assay to test the anti-tumor function of CAR-T cells in F-H. NPG mice were subcutaneously transplanted with patient-derived pancreatic tumor (PDX model). When tumor grew to about 200-300 mm^3, MPK, P4 CAR-T cells or PBS were injected intratumorally twice separated by seven days. Weight or tumor volume of mice were measured weekly. Fold change of tumor size over time **F**, the image and weight of resected tumors at 38 days after CAR-T cells injection **G,** and peripheral blood analysis of the proportion of human CD3 positive cells at 34 days after CAR-T cells injection **H** (n ≥ 4 animals per group). Statistics was performed by ANOVA with Tukey's multiple-comparisons test in C, D, F and G and by unpaired Student's t test in H. Data were represented as mean ± SD. *P < 0.05; **P < 0.01; ***P < 0.001; ****P < 0.0001.

Supplementary Figure 4. Patient recruitment and screening.

Supplementary Figure 5. Detection of MSLN expression by Immunohistochemistry. Immunohistochemistry was used to detect MSLN expression. Intensity: 0 to 3+; Grade (percent cells positive): 0 (<1%), 1 (1% to 25%), 2 (26 % to 50%), 3 (51% to 75%), or 4 (76% to 100%). Results of all patients were summarized in supplementary information,

Table S4.

Supplementary Figure 6. Representative PD-1 and TCR surface expression in MPTK-CAR-T cell infusion products.

Control cells were derived from the same donor and had the same culture conditions as MPTK-CAR-T cell product.

Supplementary Figure 7. *In vitro* expansion of MPTK-CAR-T cell products for all 17 patients enrolled.

Fold change of the cell number of MPTK-CAR-T cells after electroporation for each enrolled patient.

Supplementary Figure 8. Serum cytokine changes after MPTK-CAR-T cell infusion.

Fold changes of listed serum cytokines from baseline (on day 0 before infusion) after MPTK-CAR-T cell infusion.

Supplementary Figure 9. Four patients with baseline serous cavity effusions experienced increased effusions after MPTK-CAR-T cell infusion.

After MPTK-CAR-T cell infusion, patients MPT01, 02 experienced increased ascites; patient MPT08 experienced increased pericardial effusion and pleural effusion; patient MPT13 experienced increased pleural effusion.

Supplementary Figure 10. Flow cytometric analysis of TCR Vβ family usage after MPTK-CAR-T cell infusion.

The plot diagrams depict the quantitative analysis of the TCR Vβ repertoire of peripheral blood CD8+ (left panels) and CD4+ (right panels) T cells at day 208 in patient MPT07.

Supplementary Figure 11. Cellular kinetics of MPTK-CAR-T cells in peripheral

blood after repeat infusions.

In vivo expansion and persistence of MPTK-CAR-T cells as measured by qPCR in peripheral blood after repeat infusions (Blue arrowheads indicate the time of MPTK-CAR-T cell infusion). Day 0 is the day of the first MPTK-CAR-T cell infusion. DL, dose level.

Supplementary Figure 12. Cellular kinetics of MPTK-CAR-T cells in ascites or pericardial effusion after infusion.

In vivo expansion and persistence of MPTK-CAR-T cells as measured by qPCR in ascites or pericardial effusion (Blue arrowheads indicate the time of the second MPTK-CAR-T cell infusion). Day 0 is the day of the first MPTK-CAR-T cell infusion. PB, peripheral blood.

Supplementary Figure 13. CA19-9 levels after MPTK-CAR-T cell infusion.

A The CA19-9 concertation in peripheral blood of each patient. **B** Fold change of CA19-9 over time.

Supplementary Figure 14. Representative flow cytometry plots of circulating MPTK-CAR-T cells after infusion.

A Circulating TCR positive MPTK-CAR-T cells and TCR negative MPTK-CAR-T cells by flow cytometry 2 weeks (±1 day) after infusion in the two patients (MPT14, 17). CD2 positive cells were gated on to select the T lymphocytes population. FMO, fluorescence minus one. **B** PD-1 expression of circulating CAR+ T cells at day 13 in patient MPT14.

Supplementary Figure 15. Flow cytometric evaluation of MPTK-CAR-T cells in pericardial effusion after infusion.

TCR positive MPTK-CAR-T cells and TCR negative MPTK-CAR-T cells by flow cytometry

in pericardial effusion from patient MPT08. CD2 positive cells were gated on to select the T lymphocytes population.

Supplementary Figure 16. Proliferation of *TRAC* knock-out P4 CAR-T cells when encountering antigen *in vitro*.

A The proliferation of P4, MTK and MPTK CAR-T cells without tumor incubation. **B** The cell number of CAR-T cells (left panel), the proportion of CD3 negative and CD3 positive CAR-T cells (middle panel) and the proportion of TCR negative and TCR positive CAR-T cells (right panel) after being incubated with CRL5826-PD-L1 at E:T ratio 1:1 for 2 days. **C** The cell number of CAR-T cells (left panel), the proportion of CD3 negative and CD3 positive CAR-T cells (middle panel) and the proportion of TCR negative and TCR positive CAR-T cells (right panel) after being incubated with CRL5826-PD-L1 in E:T ratio 0.1:1 for 4 days. **D** The cell number of CAR-T cells (left panel), the proportion of CD3 negative and CD3 positive CAR-T cells (middle panels) and the proportion of TCR negative and TCR positive CAR-T cells (right panels) in each round with re-challenged with CRL5826-PD-L1 at E:T ratio 2:1 with 2 days interval.

Supplementary Tables

Supplementary Table 1. Off-target candidates genotyping

top 10 potential off-target sites and top 3 with exon hits for each sgRNA using Digenome-seq analysis

Locus name	In exon	position chromosome	mismatch sequence	Gene-editing analysis (%)	R^2
TRAC-sgRNA on target	Yes	chr14_23019528	TTCGGAACCCAATCACTGACAGG	92	0.92
offsite1	No	chr2_162200838	TAACCAGAACCCAATCACACTGG	0	1
offsite2	No	chr4_119155742	TAGGGAATCCAATTATTGACAGG	0	1
offsite3	No	chr7_157899726	TAGGAACCCAAAACACTGACTGG	0	1
offsite4	No	chr21_31543102	CAGGGAACCCAACCATTGACTGA	0	1
offsite5	No	chr3_23357613	GGTCCCCTGTAACCACTGACTGG	0	1
offsite6	No	chr11_5057956	AGAATTAGAACCCAATCAACAGG	0	1
offsite7	No	chr12_48675055	AATCTATCCCAATCACTGACTGG	0	1
offsite8	No	chr1_151691552	GCTGTCTCCCCAATTACTGACTG	0	1
offsite9	No	chr11_8738053	ACAGACACCCAACCACTGTCTGG	0	1
offsite10	No	chr6_33631947	AGGGAACCCAAACACTGGATGGG	0	1
offsite11	Yes	chr11_64721880	CTCACCATCCCAACCACTGCCTG	0	1
offsite12	Yes	chr17_9631634	GGCTCAACCCAACCACTGTCTGG	0	1
offsite13	Yes	chr22_36731741	AGTGCCCCCCAACCACTGAGCTG	0	1
PDCD1-sgRNA on target	Yes	chr2_242800943	GTCTGGGCGGTGCTACAACTGGG	87	0.91
offsite14	No	chr2_218698231	CACCTGGGCTGTGCTAGAACTGG	0	1
offsite15	No	chr16_1244458	GGCCTGGACGGTGGCACAACTCG	0	1
offsite16	No	chr12_103899414	GGGGCCTGGGCAGTGCTAACTTG	0	1
offsite17	No	chr8_134612168	CTGCTGGGCAGTGCTCAACTTGG	0	1
offsite18	No	chr9_30943866	GCCTGGGCGCTGTCACAACTCGG	0	1
offsite19	No	chr12_695889	AACTGGGCAGTGCTGAAACTGGG	0	1
offsite20	No	chr10_3030225	GGCTGGGCGATGCTATGACTCGG	0	1
offsite21	Yes	chr3_49450059	CACCTGGACGGTGATACAACCCG	0	1
offsite22	No	chr1_26478250	ATACTGGGCTGTGCTACAAGTTG	0	1
offsite23	No	chr7_135439645	TGCTGGGCATTGCTACGACTTGG	0	1
offsite24	No	chr6_169092330	GACTGGGCAGTGCTATCACTCGG	0	1
offsite25	Yes	chrX_37935897	CACTGGGCGGAGCTATAGCTTGG	0	1
offsite26	Yes	chr5_179734205	TGATGGGGCGGGGCTACAACTAT	0	1

R^2 is a measure for the reliability of the estimated values, assess the goodness of fit and indicates the

sensitivity of the analysis.

Genotyping primers for off-target analysis

PCR primers of the off-target sites:

	Forward	Reverse
offsite1	GGAGGATGGGCATTGGTTATATGC	AGCAGGCTTGTGTCCACAGATA
offsite2	CACCTGTGGTCTCAGCTAATTTGG	CTTCATCCTCCTGAGTAGCTCGAA
offsite3	CCTTGGGGATATAACCCTGTCACA	CTGATGTGACTGAGGGTACTGGAA
offsite4	ATGTGCCTAGAGTGGACACTGT	GTTGGGCAGGGGTCATTCAAAA
offsite5	GCTGTGATTCTTGCAGACCTGT	CCTTGGCTCTTGGACAGCATTT
offsite6	TACTTCTGGCCAGGCATCACA	CCATCCTGCACTGCATTGTTCT
offsite7	GACTGCTCAGTGACTATCCAAGGA	TCTGTGTTGAGTGGACCCATTACA
offsite8	AGGGAATCAAGAAGGGCACACA	TGGTGGGCAGAGAAAGTATCCT
offsite9	ACACCTGTGCTCTCATCTGAGT	GTTGCAGTAAGCCAAGATCGCT
offsite10	TGTGCCTCATGGCAATGACATC	TCCATCTACCCCCAAGAGTTTCAG
offsite11	CAACCCCCAGAACTCTAACTCCTT	TGGAGGTAGGGAAAGAAGCATACG
offsite12	ATCCAAGGAGAAACCACCAGGT	TCTCCTTCTTCCGAAACACGGA
offsite13	AGAGTCATCCCATCTGTGGCTT	AGCATTTTGGGGGAAGCAGATC
offsite14	CAGGTACGCGCATGTTTAACCA	CATGTCTCTGTTCACACTGGTCAC
offsite15	AGAGTCAAGCACAGGCCCAA	GTTGGCTCATCAGGAAAGCGG
offsite16	TCTGACCCCATAGTGTTGGGTT	TAGGAAAAGCTCCAGGGGTTCA
offsite17	AGGACTGCCACAAGTAAGGGTT	AGCAGCAGGCACATTCTTCATC
offsite18	TAAAGCAGATGGGAGCTGGGAA	AACGTCTGTCTGAGTCTGGCTT
offsite19	TTGGGATTTCTGGTGACCAGGA	ACCATCTGCTCACACTAAGGGT
offsite20	TCCTCTCTCTTCCTTTCCTCCTCT	AAGCCTCCACTGACTGAAGGTT
offsite21	CGTGCGCGGTTTTAATACTCCT	GTGAAGTCTGGTGAGGGTCCTAA
offsite22	GACATCTGCTCTCACACCTAATTCC	CGATAGAGCAAGACTCTGTCTCTGA
offsite23	AGCAACTTGGAAGGCTGAATGG	GAACAGTGCCGCAAGACTGTAA
offsite24	TCCATCCCCTTCTTTCTGCACA	GGTATGGTTGAACGGCACGATT
offsite25	CTATTCCTGCAAACAGGCCCAA	TGCAGGTGACATAGGCAATTCC
offsite26	AAGCAGCTCATGAGGTCTGAGT	GTTCACACAACACGAATCCCCA

Oligo nucleotides for sequencing:

	Forward	Reverse
offsite1	ACTAGGCTATTTTACATTAG	TAAATGGGCAGCAGACCTGC
offsite2	AACCTGAGCCCATGGAGGTTG	CACCTGCCAACAAGCCCAGC
offsite3	TCCCCGAAGGTGATAGAGTG	GCTTTCAGCACACCAGGGAC
offsite4	TTTTCAGCTGCTTAGAGTGC	TTGGATCTGATTTTACAGGC
offsite5	TGTTAAGATCTGGAGGAATTC	GAATTGCAAGTGTGGCAGC
offsite6	GAAATCGATGTTTCTTATAC	GATCAGATGAAGCAGTTGC
offsite7	GGATTTATTAAAAGAGTGAG	ACAATTCAAAAATATTCATG

offsite8	ACATCTTGAAAGTAACTTTC	CTCCTAGAGAGAAACTTTCC
offsite9	AATTTTGCGGGGGTCCTCC	AAGATTTACCCTCTAATTAG
offsite10	TTTGAGCTGGACCCCACCAC	GTGACTCAATTAACTCTCCC
offsite11	TTCCACCAATTTGTAAAGTTG	CTCTTTAAGGATGCATCCAC
offsite12	CGAGTTGGTGGAGTACTTGG	CAGAAGGGTGAGTGTCCCC
offsite13	GCGCTCCTTCCTCAGCTGCC	CCTAAACTTGTCTGAGAGC
offsite14	GGCGCAGGTGGGCCCGTG	CTGCGGCCATGTGTGGCTC
offsite15	GGTGGGGGCGGCCACCGGGC	GAGCCCTCGGTGGGCTGGCC
offsite16	GAAGTAGTTTCAGTCACAGG	AAGATCAGCCATTTGGAAAC
offsite17	TGGGGCCTTGAACAGGTTGG	TGAGGAGAAGCATGGAGCAG
offsite18	CCCCTTTCTGAGTTGGTGCG	GAAGAGAGAAAGTGCCATCC
offsite19	AAAGCCAGTGGCTGCTTCAT	TCCATCCAAACTTCAACTCC
offsite20	TCTCCCCCACCCTTCTTTCTGG	GCAGCAGTCAGATTTTTGAC
offsite21	CTTCACGCACGCGAAGTAGG	GTGAGAGTTCTGAGGAACAG
offsite22	AGAAACACATGCTTAACAAC	ATGTGGTCTCTCAAAATATC
offsite23	TAAGCCTGGGAGATCAAGGC	TGGAGACAGAACTCCAAGTC
offsite24	ACCATGCATTCCATGTCTTG	TGTGTGTCAGAAGGCAGGCC
offsite25	GGTGACAGTGCTGAGTTCTC	TACGTGCTTTCCATATCAAC
offsite26	GGCAGTGCCTGGACAGCCGG	GGAGCCCAAGTTATTTCCTG

Supplementary Table 2. Clinical outcome after MPTK-CAR-T cell infusion

Patient No.	Diagnosis	CAR-T cells/kg × weight(kg) (Dose level)	Disease response(3-4 w)	Progression free survival(months from first MPTK-CAR-T infusion)	Survival(months from first MPTK-CAR-T infusion)	Outcome
MPT 01	Pancreatic Cancer	$1.8\times10^5\times65$ (DL1)	PD	0.8	1.1	DOD
MPT 02	Biliary Tract Cancer	$2.2\times10^5\times75$ (DL1)<br>$7.7\times10^5\times79$ (DL2)	SD	0.9	1.4	DOD
MPT 04	Gastric Cancer	$2.3\times10^5\times46$ (DL1)<br>$6.6\times10^5\times47$ (DL2)<br>$3.2\times10^6\times43$ (DL3)	SD	4.7	5.2	DOD
MPT 05	Pancreatic Cancer	$2.7\times10^5\times46$ (DL1)	PD	0.8	1.5	DOD
MPT 06	Biliary Tract Cancer	$4.2\times10^5\times50$ (DL2)	PD	0.8	1.2	DOD
MPT 07	Tubal Cancer	$8.3\times10^5\times72$ (DL2)<br>$2.7\times10^6\times70$ (DL3)<br>$2.7\times10^6\times69$ (DL3)	PD	0.9	12.7	DOD
MPT 08	Esophagus Cancer	$9.0\times10^5\times43$ (DL2)<br>$3.3\times10^6\times45$ (DL3)	PD	0.9	2.3	DOD
MPT 09	Ovarian Cancer	$8.1\times10^5\times74$ (DL2)<br>$3.1\times10^6\times75$ (DL3)	PD	0.7	21.0	Alive
MPT 10	Pancreatic Cancer	$1.8\times10^6\times68$ (DL3)	PD	0.9	1.2	DOD
MPT 11	Pancreatic Cancer	$2.5\times10^6\times60$ (DL3)	PD	0.9	1.8	DOD
MPT 12	Pancreatic Cancer	$2.3\times10^6\times66$ (DL3)<br>$5.1\times10^6\times62$ (DL4)	SD	2.1	4.9	DOD
MPT 13	Cervical Cancer	$2.1\times10^6\times58$ (DL3)	SD	1.3	3.1	DOD
MPT 14	Triple-Negative Breast Cancer	$5.4\times10^6\times63$ (DL4)<br>$5.9\times10^6\times62$ (DL4)	SD	2.6*	19.3	Alive
MPT 16	Biliary Tract Cancer	$6.9\times10^6\times70$ (DL4)<br>$7.2\times10^6\times66$ (DL4)	SD	1.7	3.0	DOD
MPT 17	Pancreatic Cancer	$11.0\times10^6\times75$ (DL4)	SD	0.7*	15.7	Alive

Abbreviation: PD, progressive disease; SD, stable disease; DOD, died of disease; DL, Dose level.

*, no progression, receiving other therapy

Supplementary Table 3. Treatment history of all 15 patients infused

Patient No.	Prior Therapy
MPT01	surgery; (gemcitabine, nab-paclitaxel)×2
MPT02	transarterial chemoembolization; (gemcitabine, oxaliplatin)×6; gemcitabine, nab-paclitaxel; (gemcitabine, cisplatin, nivolumab)×2
MPT04	surgery; capecitabine; (oxaliplatin, fluorouracil, leucovorin) ×6; (oxaliplatin, epirubicin hydrochloride, fluorouracil, nivolumab)×3; (decitabine, paclitaxel, cisplatin, nivolumab)×4; (decitabine, nab-paclitaxel, cisplatin)×2
MPT05	(gemcitabine, TS-1)×2
MPT06	surgery; gemcitabine, cisplatin
MPT07	(paclitaxel, carboplatin)×2; surgery; (paclitaxel, carboplatin)×8; surgery; (decitabine, paclitaxel, cisplatin)×5; (bevacizumab, gemcitabine, carboplatin)×4; bevacizumab, docetaxel, lobaplatin; (paclitaxel, carboplatin, nivolumab)×4; liposomal adriamycin, cisplatin, investigational anti-PD-1 antibody; (nab-paclitaxel, cisplatin, investigational anti-PD-1 antibody) ×2; carboplatin, vinorelbine; bevacizumab, paclitaxel, cisplatin
MPT08	surgery; radiotherapy; paclitaxel×4; (cisplatin, fluorouracil)×2; radiotherapy; TS-1×3; radiotherapy, cisplatin; cisplatin×3; radiotherapy; investigational anti-PD-1 antibody×3; gemcitabine×6; irinotecan
MPT09	surgery; paclitaxel; (paclitaxel, lobaplatin)×2; surgery; (paclitaxel, carboplatin)×5; radiotherapy; nivolumab×2; (nab-paclitaxel, cisplatin)×2; (nab-paclitaxel, cisplatin, nivolumab)×6; nivolumab; bevacizumab, paclitaxel, cisplatin
MPT10	(gemcitabine, nab-paclitaxel)×3
MPT11	gemcitabine, nab-paclitaxel
MPT12	gemcitabine; TS-1; radiotherapy, gemcitabine; radiotherapy, TS-1
MPT13	surgery; (paclitaxel, carboplatin)×6; radiotherapy; (gemcitabine, lobaplatin)×4
MPT14	(pirarubicin, paclitaxel)×2; surgery; (pirarubicin, paclitaxel)×6; (lobaplatin, vinorelbine)×2; (gemcitabine, cisplatin)×2; (decitabine, investigational anti-PD-1 antibody)×3; (bevacizumab, liposomal adriamycin, carboplatin)×6; surgery; radiotherapy
MPT16	surgery, radiotherapy; gemcitabine, cisplatin
MPT17	surgery; (gemcitabine; TS-1)×6; nab-paclitaxel×2; gemcitabine, nab-paclitaxel

Supplementary Table 4. Results of MSLN Immunohistochemistry

Patient No.	Intensity	Percentage of MSLN expressing tumor cells	Grade
MPT01	2+	70%	3
MPT02	0	0%	0
MPT03	2+	10%	1
MPT04	0	0%	0
MPT05	3+	90%	4
MPT06	0	0%	0
MPT07	3+	90%	4
MPT08	2+	10%	1
MPT09	2+	80%	4
MPT10	2+	80%	4
MPT11	2+	80%	4
MPT12	0	0%	0
MPT13	2+	90%	4
MPT14	1+	90%	4
MPT15	2+	30%	2
MPT16	3+	98%	4
MPT17	1+	10%	1

Abbreviation: MSLN, mesothelin.

Intensity: 0, 1+, 2+, 3+.

Grade (percent cells positive): 0, (<1%), 1 (1% to 25%), 2 (26 % to 50%), 3 (51% to 75%), or 4 (76% to 100%).

Supplementary Table 5. Results of PD-L1 Immunohistochemistry

Patient No.	Intensity	Percentage of PD-L1 expressing tumor cells	Grade
MPT01	2+	30%	2
MPT02	0	0%	0
MPT03	0	0%	0
MPT04	0	0%	0
MPT05	0	0%	0
MPT06	0	0%	0
MPT07	0	0%	0
MPT08	0	0%	0
MPT09	2+	20%	1
MPT10	0	0%	0
MPT11	1+	1%	1
MPT12	1+	2%	1
MPT13	0	0%	0
MPT14	NA	NA	NA
MPT15	NA	NA	NA
MPT16	0	0%	0
MPT17	1+	30%	2

Abbreviation: NA, not assessed.

Intensity: 0, 1+, 2+, 3+.

Grade (percent cells positive): 0, (<1%), 1 (1% to 25%), 2 (26 % to 50%), 3 (51% to 75%), or 4 (76% to 100%).

Supplementary Table 6. PD-1 and TCR expression in MPTK-CAR-T cell products and control cells

Patient No.	Control cells		MPTK-CAR-T cell products	
	PD-1+ (%)	TCR+ (%)	PD-1+ (%)	TCR+ (%)
MPT01	66.0	99.6	2.2	0.3
MPT02	24.4	99.6	0.2	0.2
MPT03	15.5	99.7	0.5	0.2
MPT04	31.2	99.4	0.5	0.1
MPT05	34.4	99.6	0.7	0.1
MPT06	17.1	99.8	0.6	0.2
MPT07	5.6	99.7	0.1	0.2
MPT08	10.0	91.9	0.4	4.0
MPT09	4.6	99.8	1.3	1.4
MPT10	16.0	99.4	1.1	2.3
MPT11	11.7	99.6	0.8	2.7
MPT12	9.8	98.2	0.9	1.4
MPT13	5.1	99.8	0.8	3.8
MPT14	7.0	99.5	0.9	1.8
MPT15	5.3	99.9	0.7	3.1
MPT16	67.3	99.2	4.0	1.2
MPT17	36.6	98.2	3.0	2.8

Control cells were derived from the same donor and had the same culture conditions as MPTK-CAR-T cell product.
