# Supplementary material for: Phase I study of CAR-T cells with PD-1 and TCR disruption in mesothelin-positive solid tumors
Source: Cell Mol Immunol. 2021 Aug 11;18(9):2188–98. doi: 10.1038/s41423-021-00749-x (PMC8429583; doi:10.1038/s41423-021-00749-x)

Supplementary Figure 1. *PDCD1* knock-out P4 CAR-T cells exhibited superior anti-tumor efficiency *in vitro*

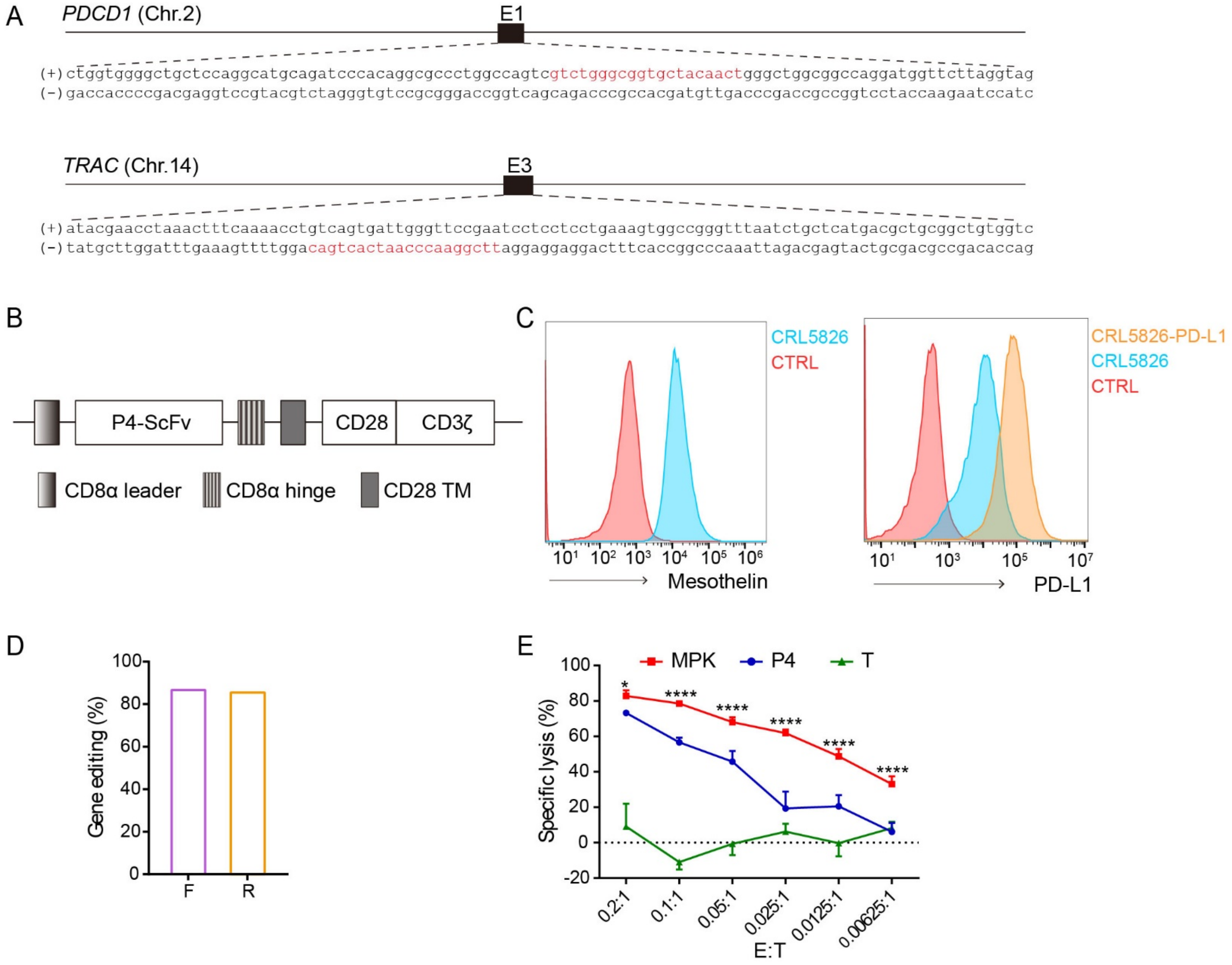

Supplementary Figure 2. Characterization of *PDCD1*/*TRAC* double knock-out P4 CAR-T cells

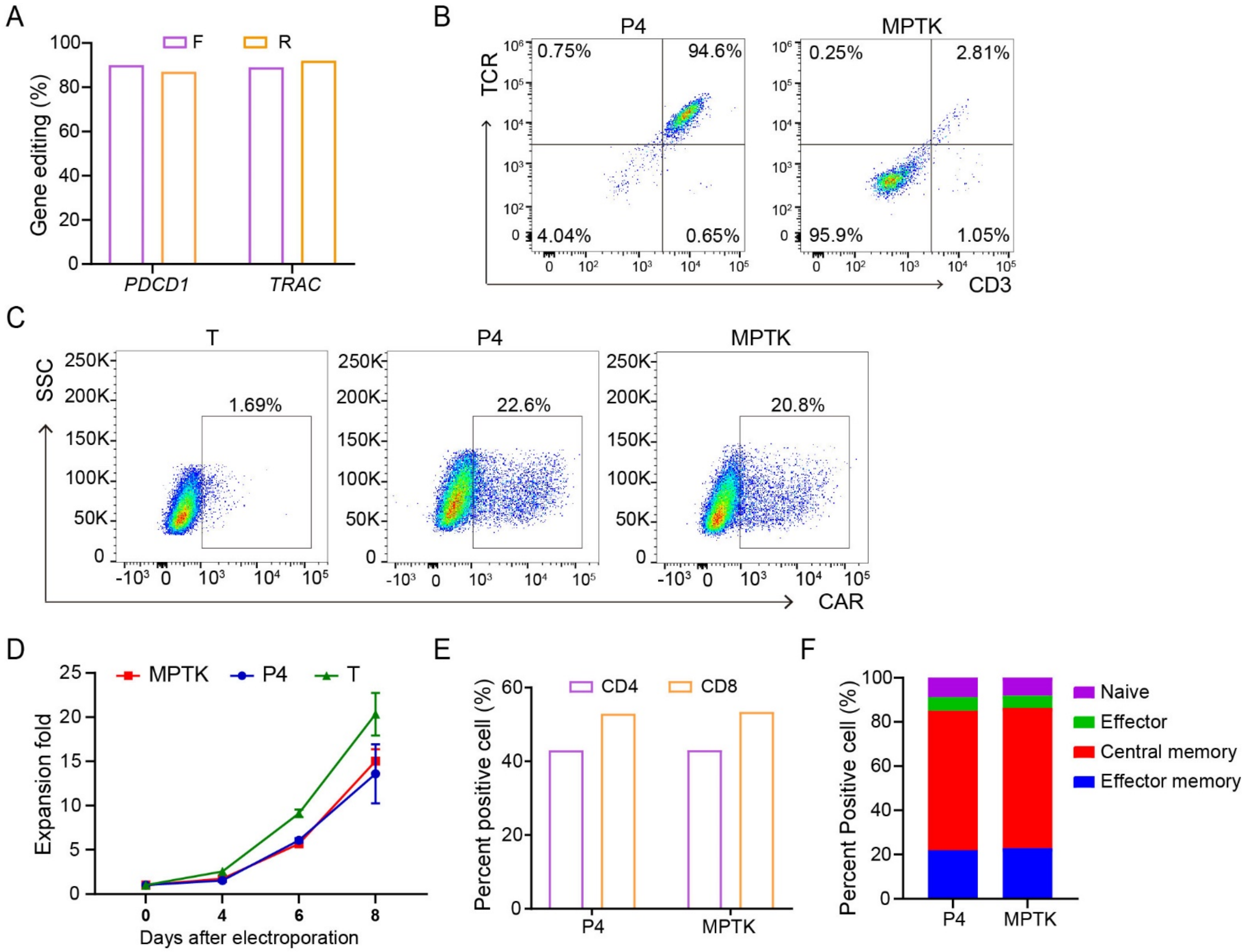

Supplementary Figure 3. *PDCD1* knock-out P4 CAR-T cells exhibited superior anti-tumor efficiency *in vivo*

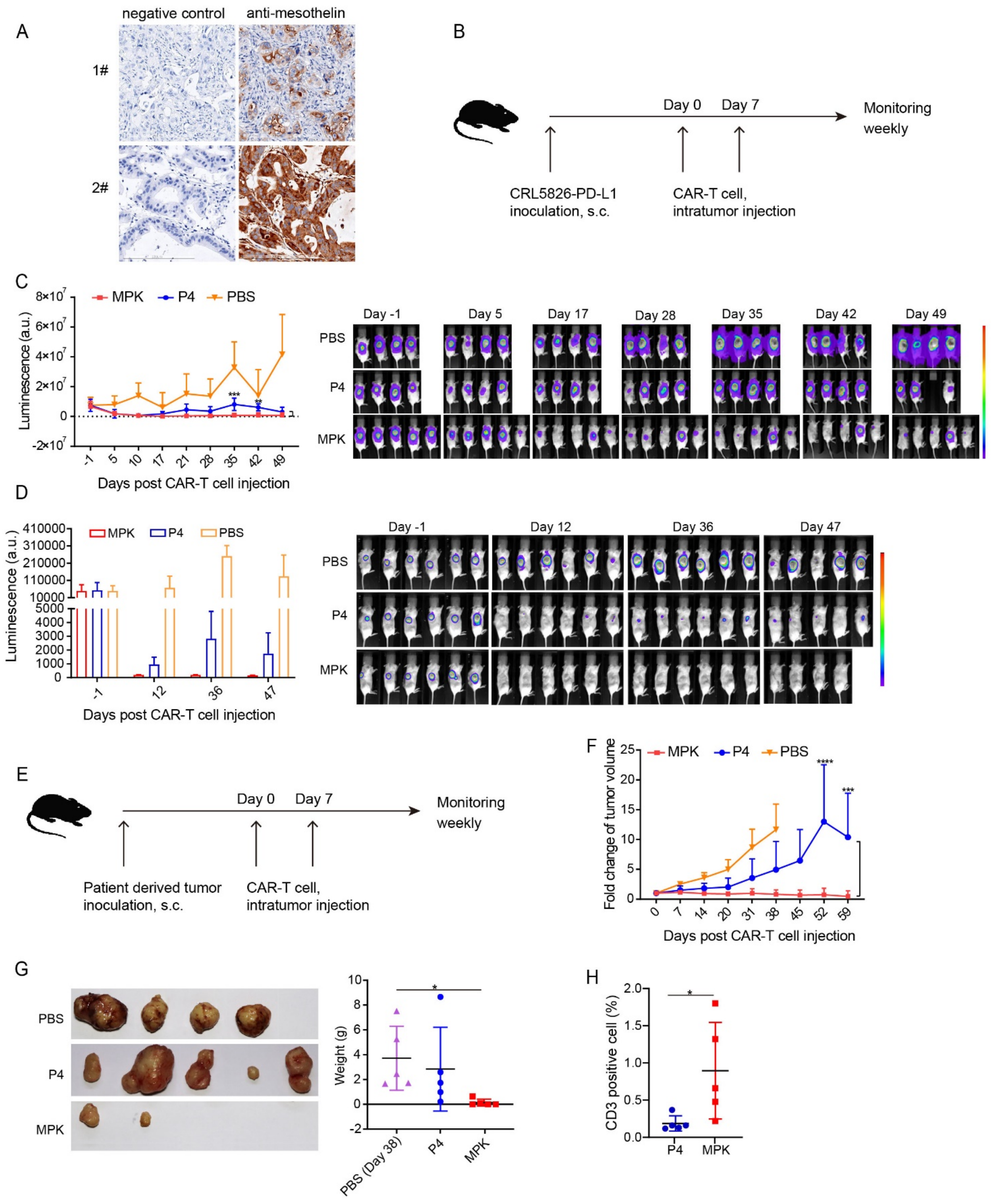

Supplementary Figure 4. Patient recruitment and screening

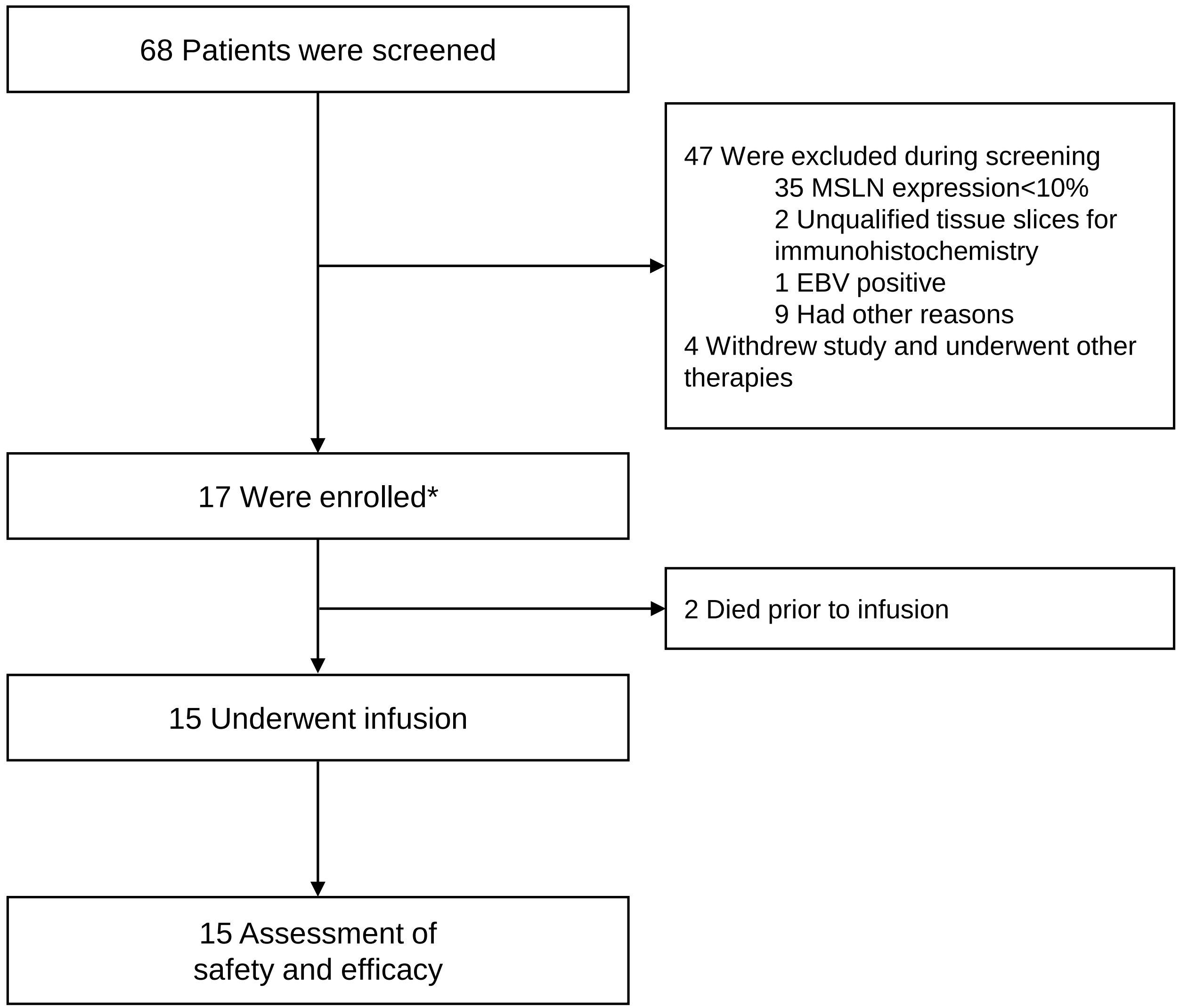

*Four patients with lower than 1% MSLN expression were enrolled in the compassionate protocol, and all received infusion.

Supplementary Figure 5. Detection of MSLN expression by Immunohistochemistry

H&E | MSLN: Intensity 2+;Grade 4 | Negative Control | Positive Control

MPT09

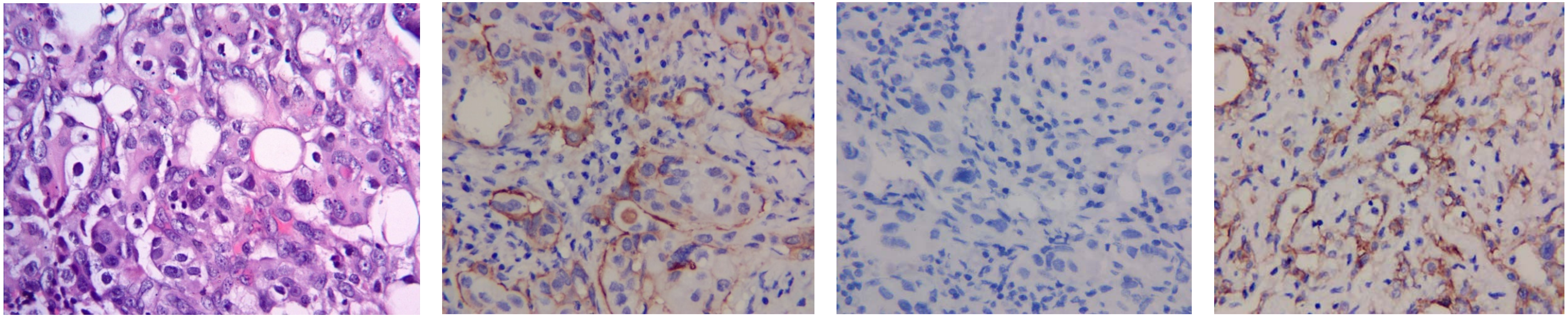

Supplementary Figure 6. Representative PD-1 and TCR surface expression in MPTK-CAR-T cell infusion product

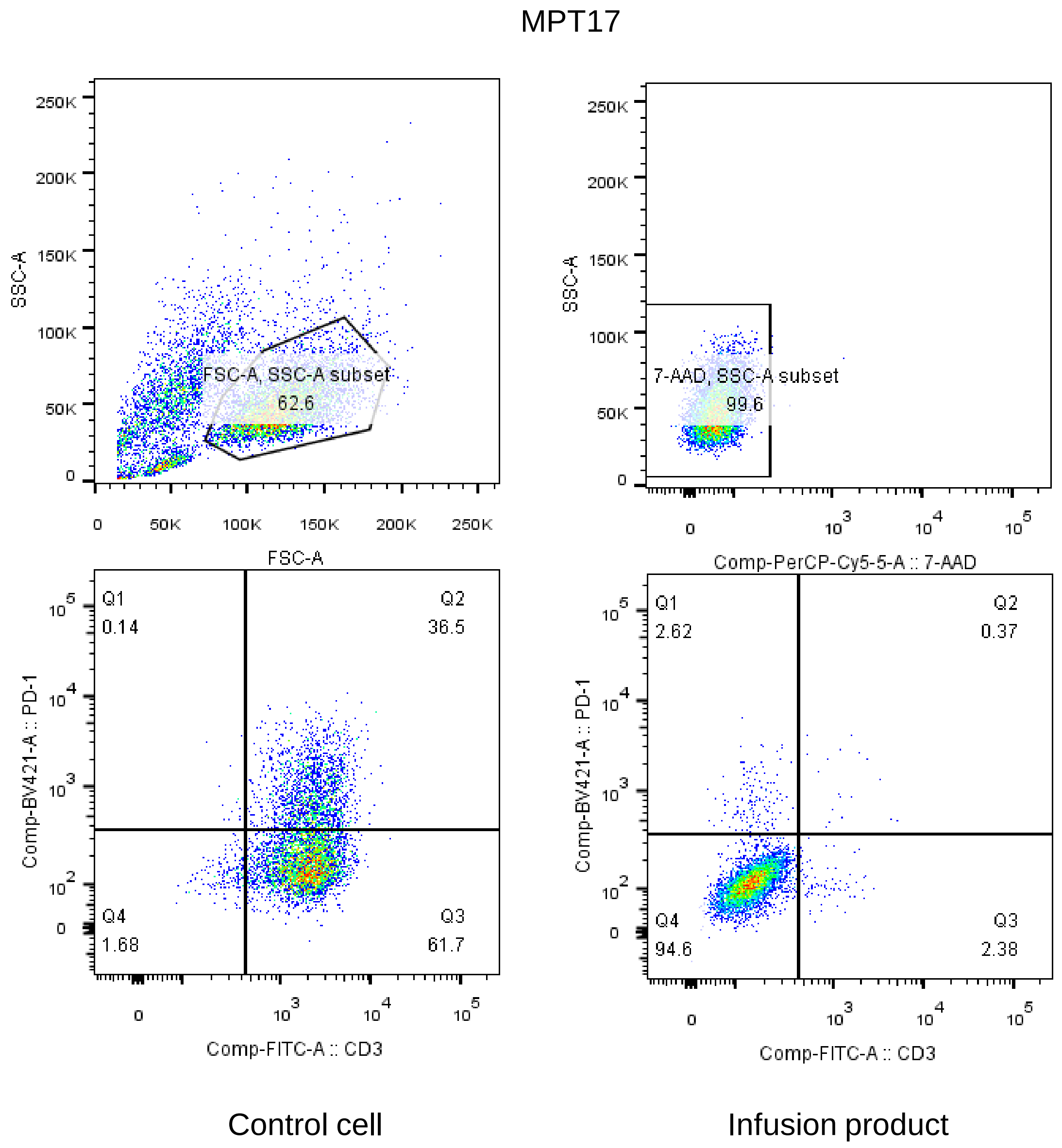

Supplementary Figure 7. *In vitro* expansion of MPTK-CAR-T cell products for all 17 patients enrolled

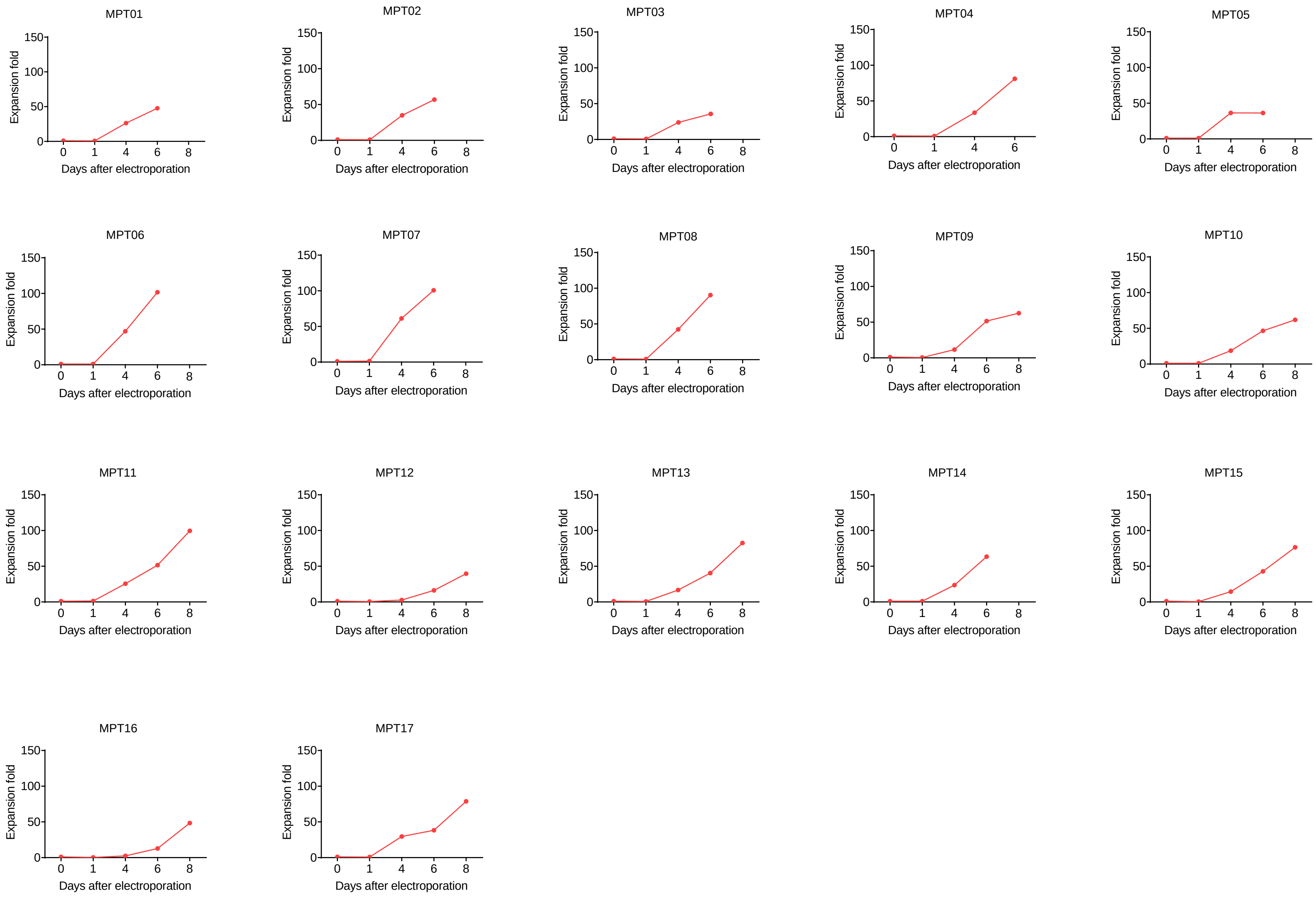

Supplementary Figure 8. Serum cytokine changes after MPTK-CAR-T cell infusion

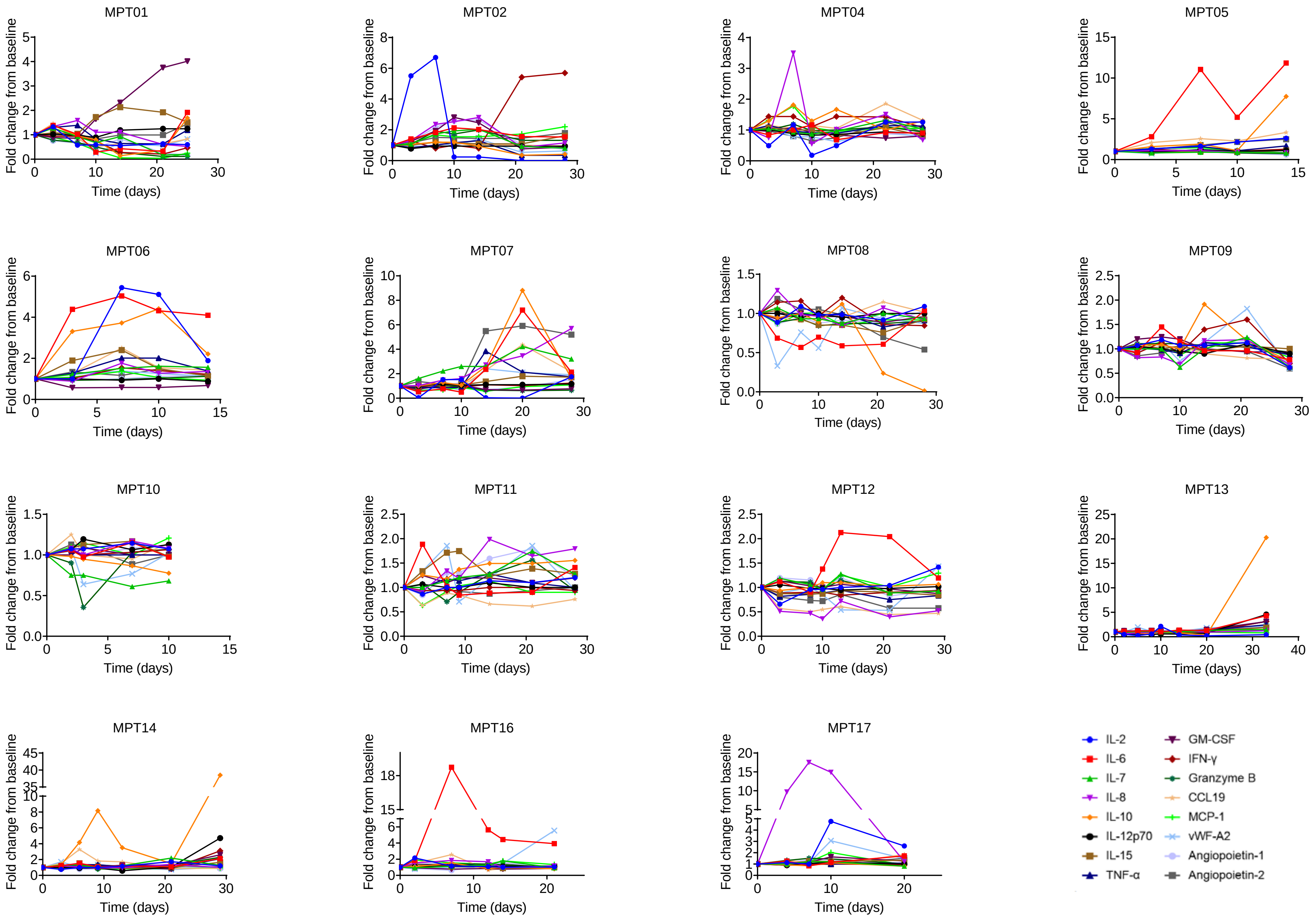

Supplementary Figure 9. Four patients with baseline serous cavity effusions experienced increased effusions after MPTK-CAR-T cell infusion

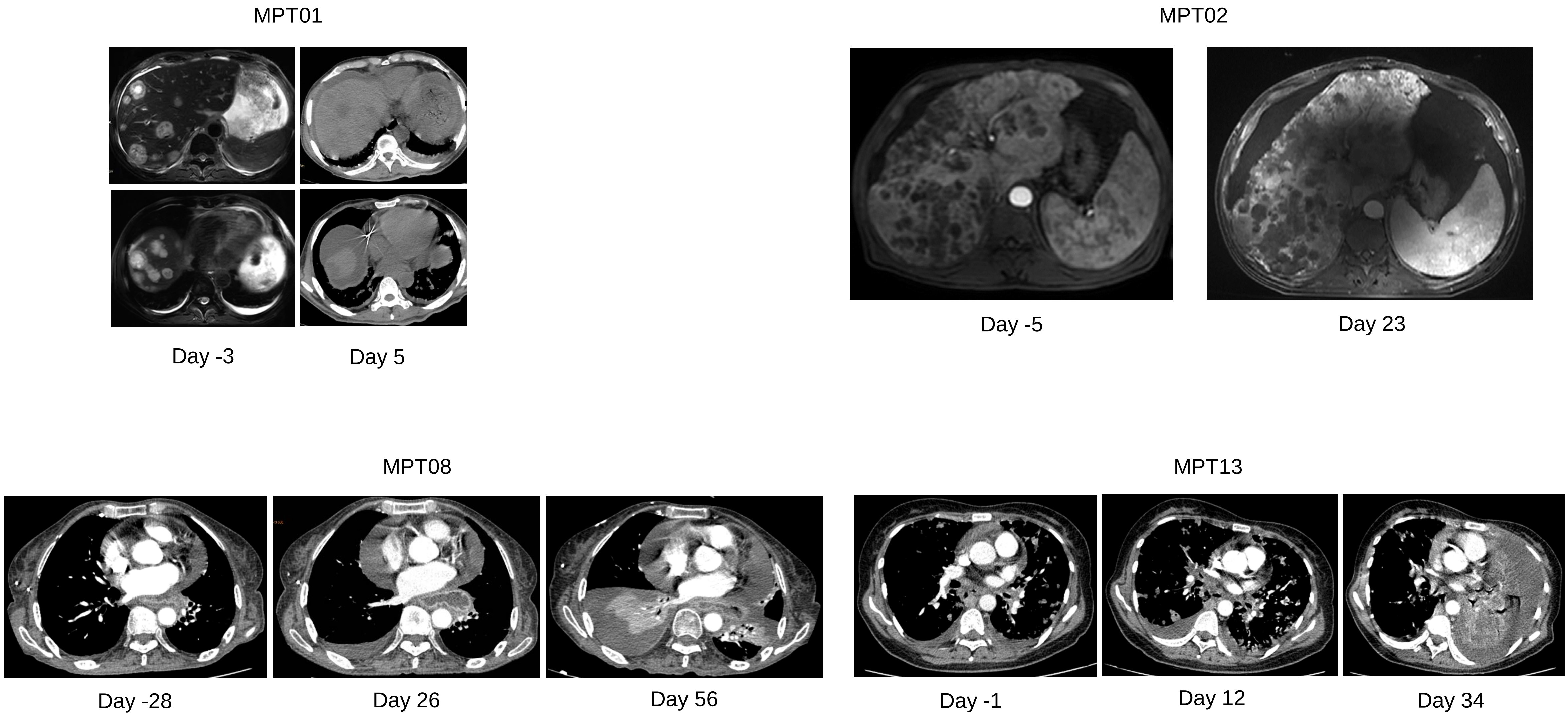

Supplementary Figure 10. Flow cytometric analysis of TCR Vβ family usage after MPTK-CAR-T cell infusion

MPT07, day 208

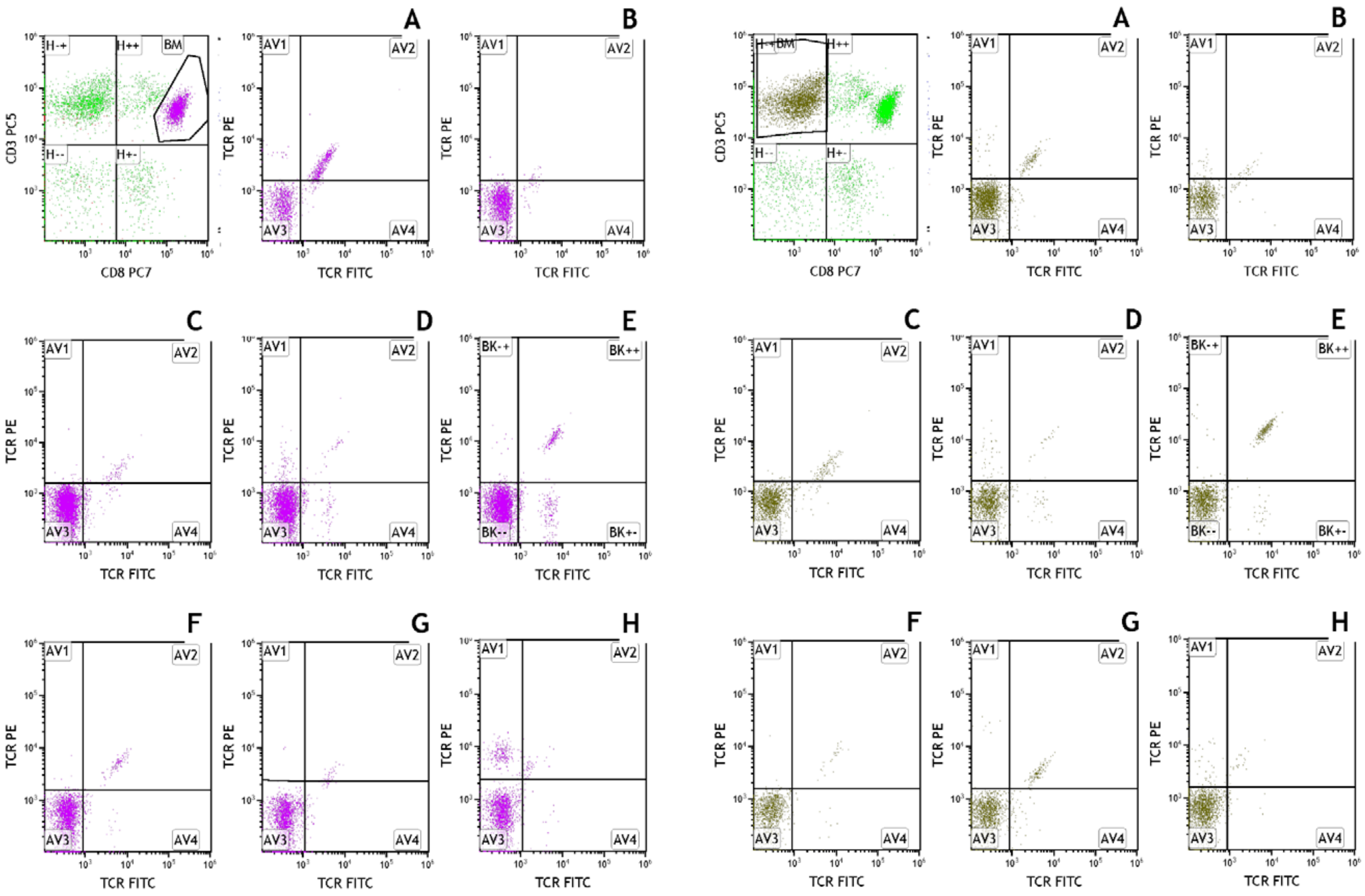

A	Vb5.3	PE
	Vb7.1	PE+FITC
	Vb3	FITC
B	Vb9	PE
	Vb17	PE+FITC
	Vb16	FITC
C	Vb18	PE
	Vb5.1	PE+FITC
	Vb20	FITC
D	Vb13.1	PE
	Vb13.6	PE+FITC
	Vb8	FITC
E	Vb5.2	PE
	Vb2	PE+FITC
	Vb12	FITC
F	Vb23	PE
	Vb1	PE+FITC
	Vb21.3	FITC
G	Vb11	PE
	Vb22	PE+FITC
	Vb14	FITC
H	Vb13.2	PE
	Vb4	PE+FITC
	Vb7.2	FITC

Supplementary Figure 11. Cellular kinetics of MPTK-CAR-T cells in peripheral blood after repeat infusions

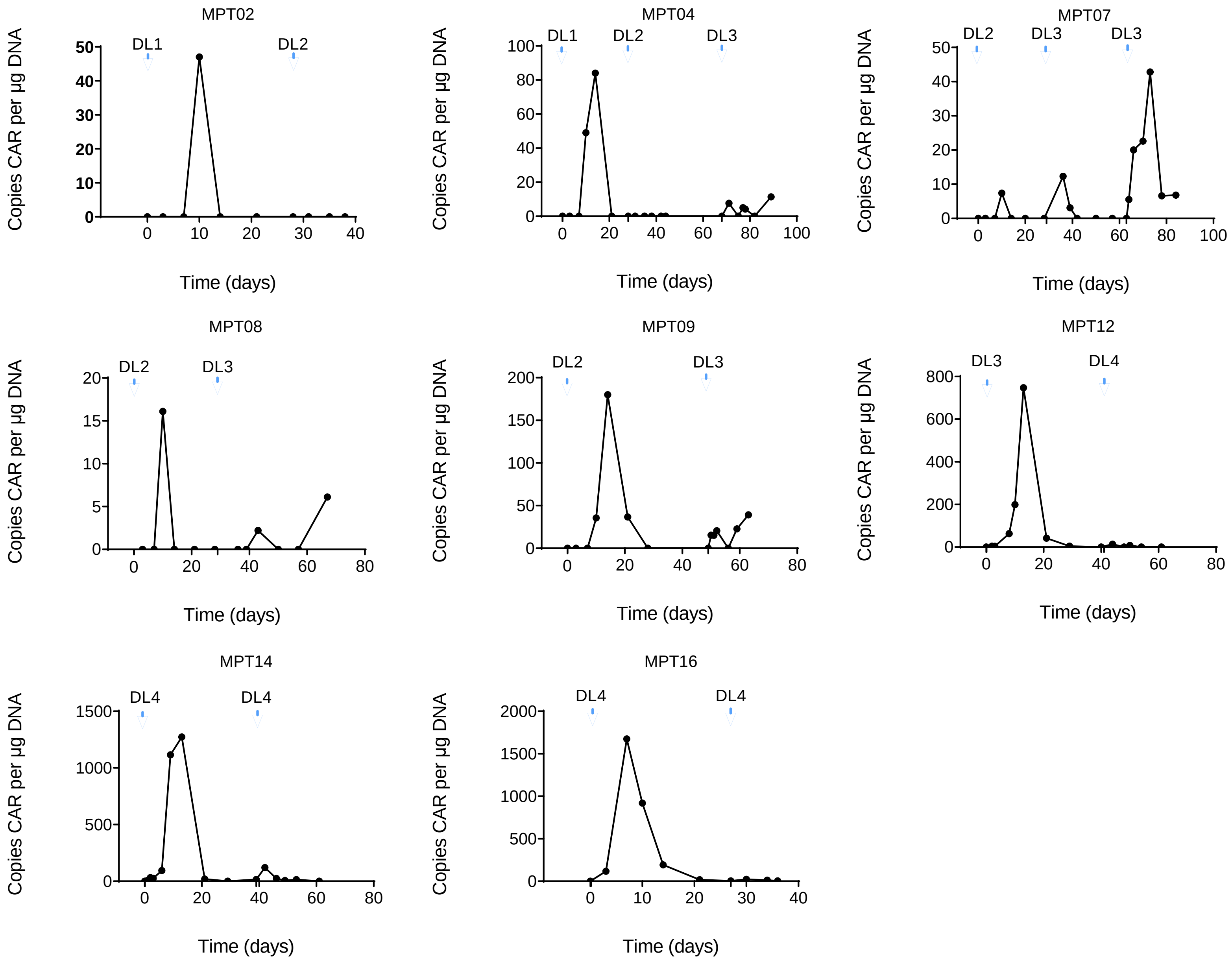

Supplementary Figure 12. Cellular kinetics of MPTK-CAR-T cells in ascites or pericardial effusion after infusion

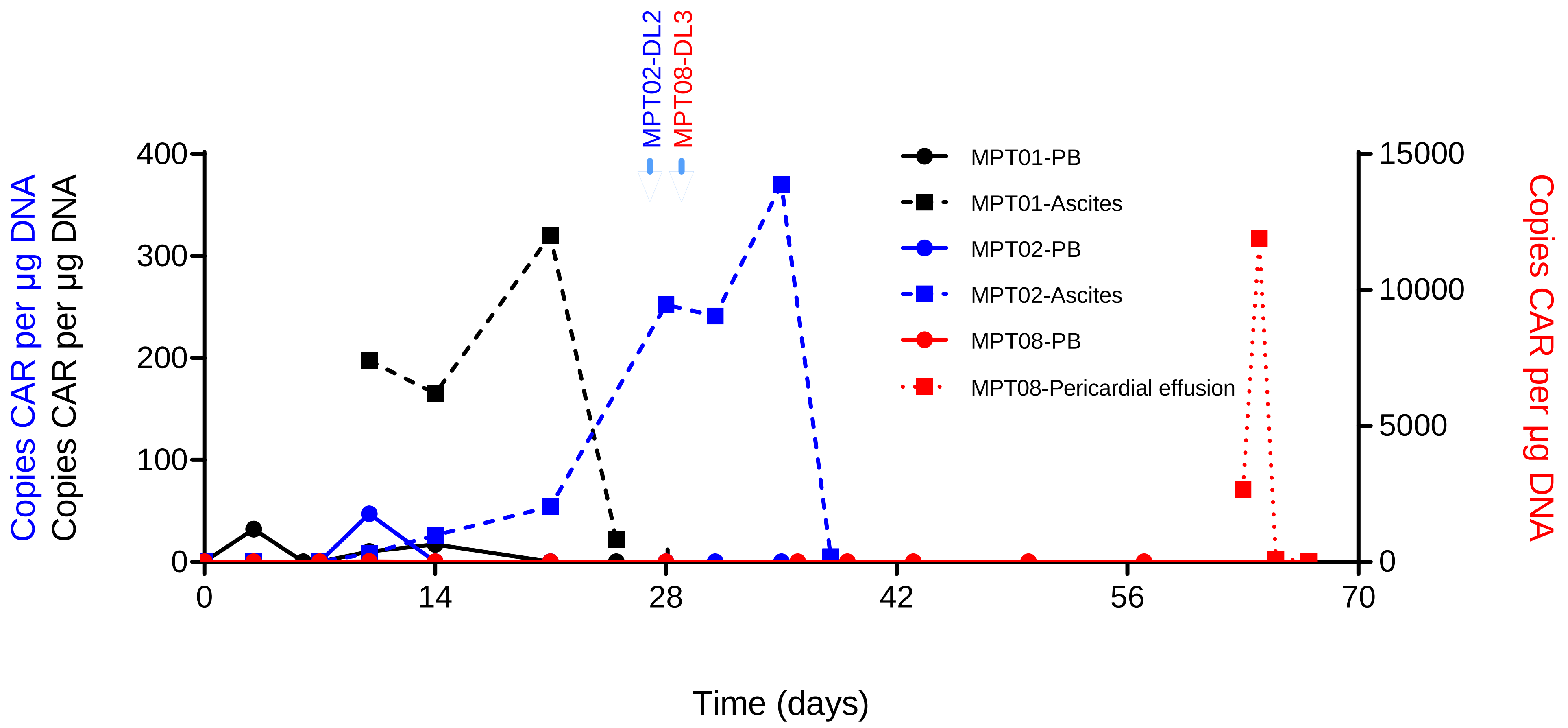

Supplementary Figure 13. CA19-9 levels after MPTK-CAR-T cell infusion

A

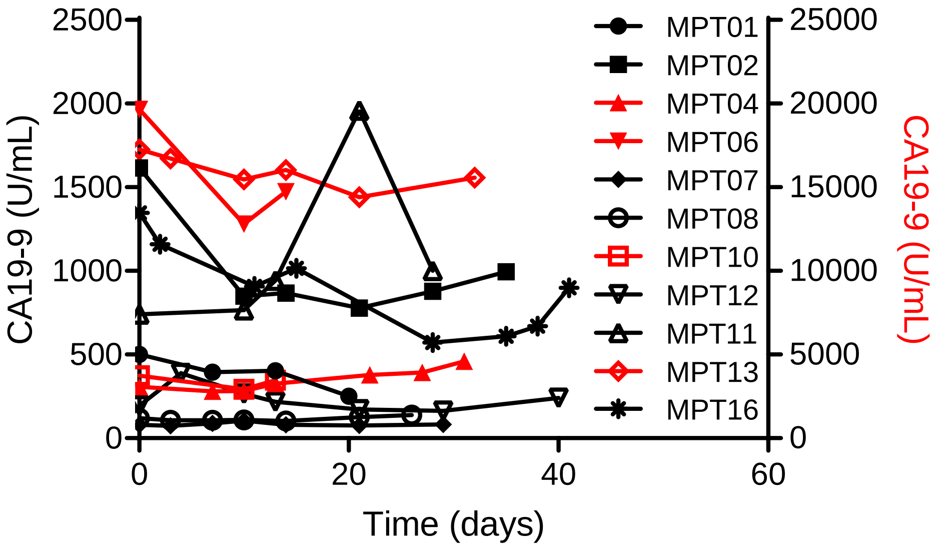

B

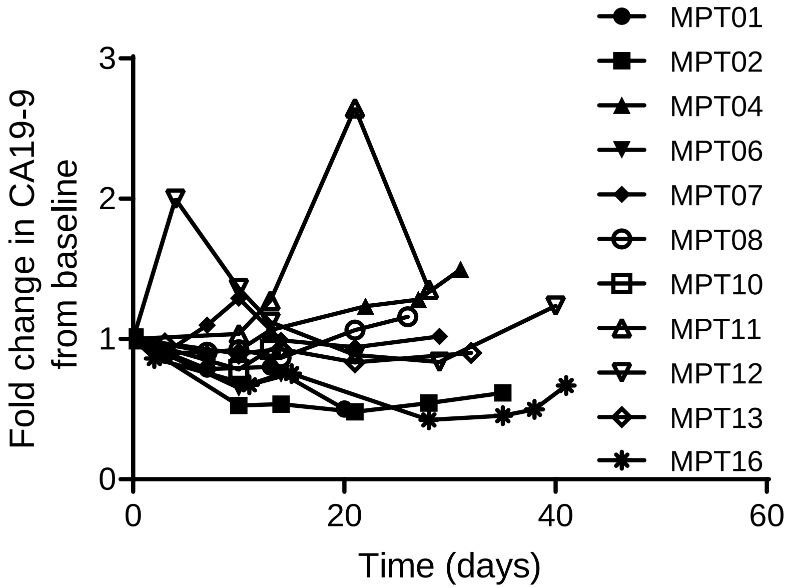

Supplementary Figure 14. Representative flow cytometry plots of circulating MPTK-CAR-T cells after infusion

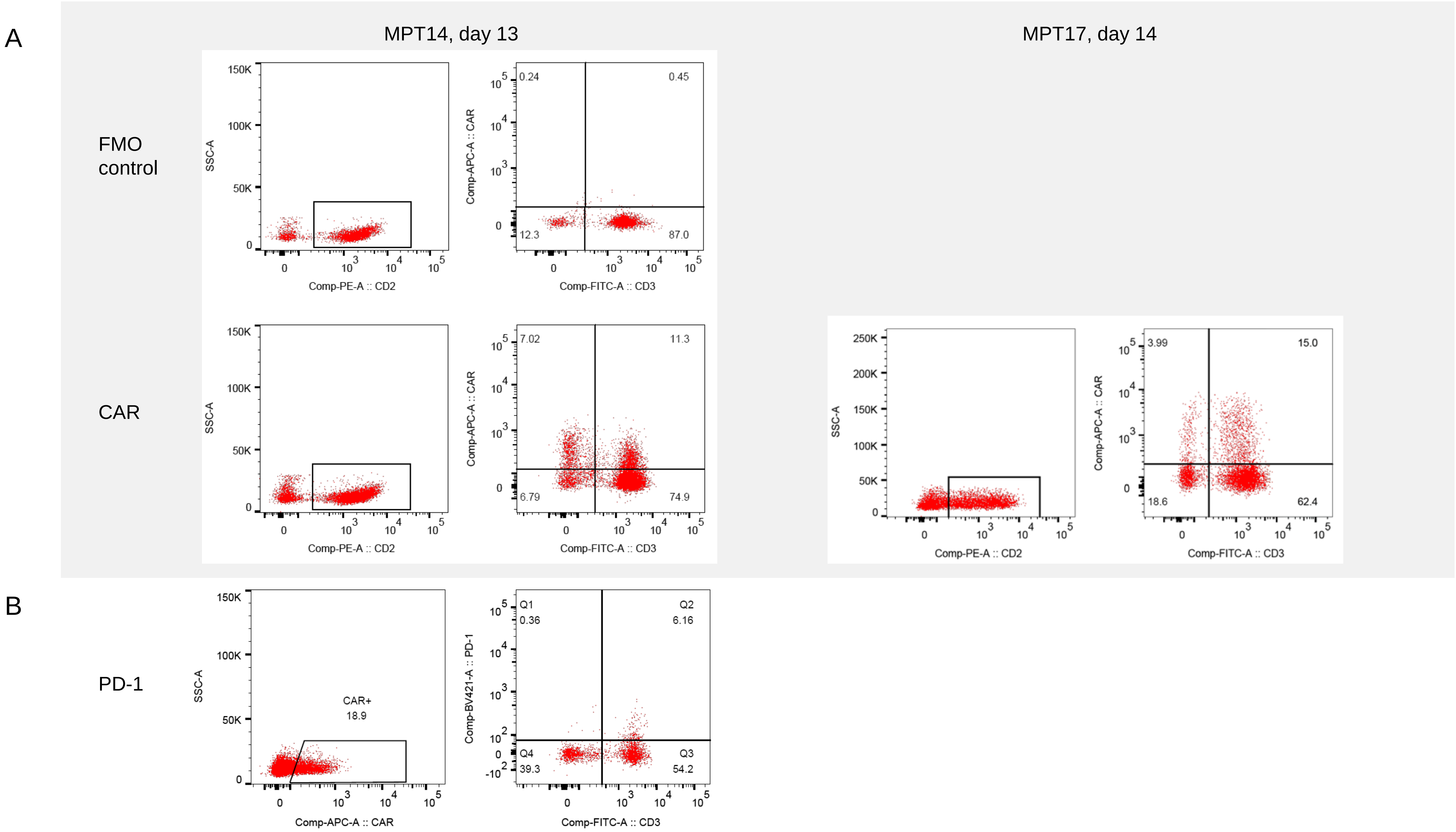

Supplementary Figure 15. Flow cytometric evaluation of MPTK-CAR-T cells in pericardial effusion after infusion

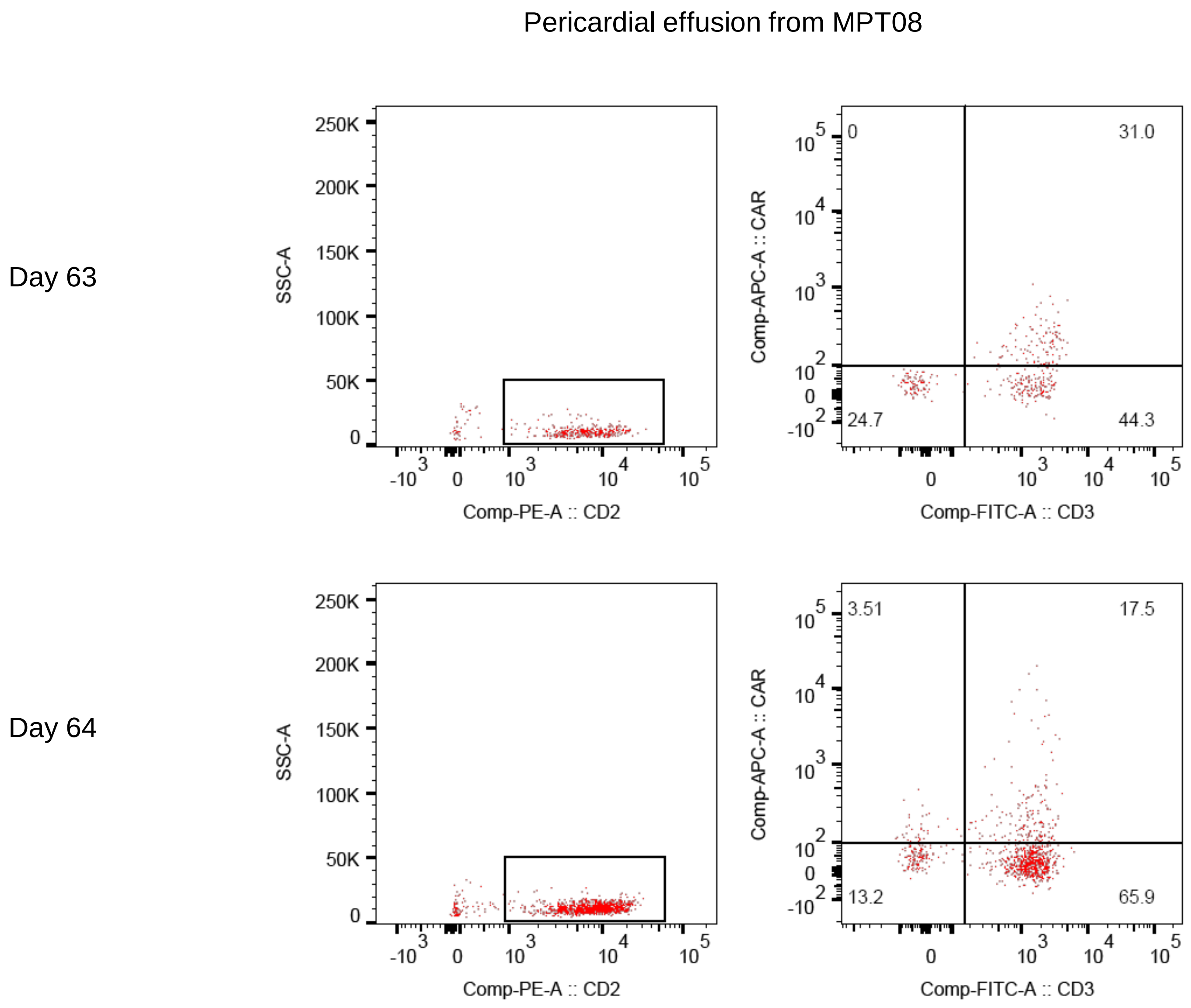

Supplementary Figure 16. Proliferation of *TRAC* knock-out P4 CAR-T cells when encountering antigen *in vitro*

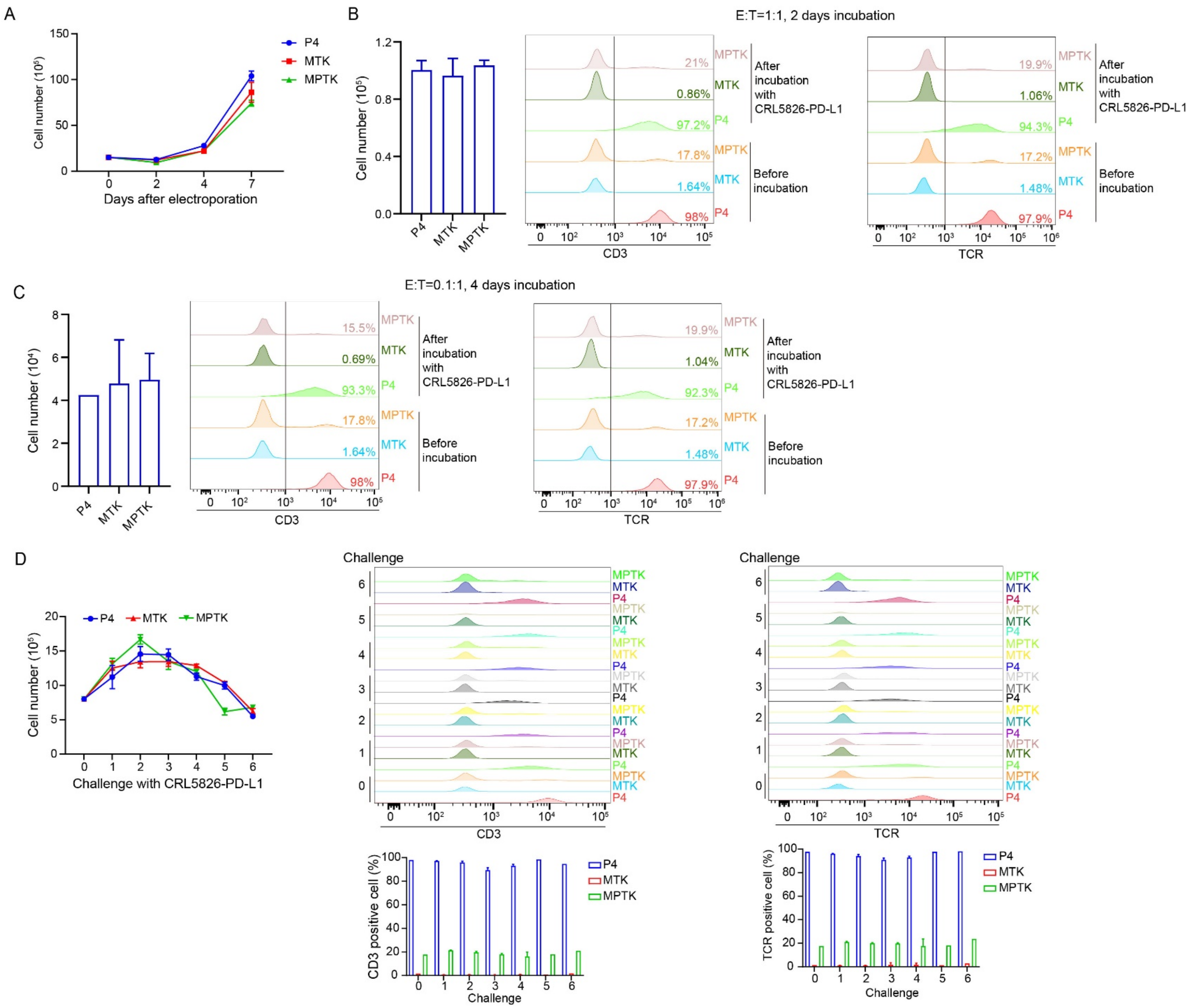

Supplement: Supplementary file 2 — Supplementary figure [file 41423_2021_749_MOESM2_ESM.pdf]
